# Supplementary material for: GenoBERT: A Language Model for Accurate Genotype Imputation
Source: ArXiv. 2026 Mar 31:arXiv:2604.00058v1. Preprint. [Version 1] (PMC13148108)
Supplement: 1 [file NIHPP2604.00058V1-supplement-1.pdf]

Supplementary material for the paper:

GenoBERT: A Language Model for Accurate Genotype Imputation

Table of Contents

OVERVIEW 30
ABLATION STUDIES 30
WEIGHT INITIALIZATION STRATEGIES 32
ON CHOOSING GENOTYPE SEGMENT LENGTH 34
COMPREHENSIVE RESULTS 35
Models' performance on LOS/mixed populations cohort 35
Models' performance on LOS/AA cohort 41
Models' performance on LOS/CA cohort 46
Models' performance on 1KGP/mixed populations cohort 51
Models' performance on 1KGP/EUR cohort 56
Models' performance on 1KGP/AFR cohort 58
Models' performance on 1KGP/AMR cohort 60
Models' performance on 1KGP/SAS cohort 62
Models' performance on 1KGP/EAS cohort 64
DATASETS SPECIFICATIONS 66

Overview

This supplement provides ablation study, GenoBERT's weight initialization details, the determination of proper segmentation hyperparameter, extended cohort-specific evaluations supporting the findings presented in the main manuscript and dataset specifications. Performance metrics include accuracy (concordance rate), r^2, precision, recall, and F_1-score (macro), evaluated across varying missingness levels, allele frequency bins, and population cohorts.

The analyses are based on three datasets:

- Louisiana Osteoporosis Study (LOS) – chromosome 22
- 1000 Genomes Project (1KGP) – chromosome 22

Models were evaluated under simulated genotype missingness levels of 5%, 15%, and 25%, with results stratified by minor allele frequency (MAF) bins (e.g., 0.1%≤ MAF ≤ 0.5%, etc.).

Ablation studies

To determine the optimal configuration, a systematic series of ablation experiments were conducted.

Alation I: Component-Wise Ablation

Ablation I is performed on two cohorts: the 1KGP/EUR cohort, with training/validation sample sizes of 417/51 and 120,577 SNPs, and the LOS/AA cohort, with 2479/309 samples and 307,599 SNPs.

This ablation study evaluates the contribution of two optional architectural components:

- RGPB: whether the Relative Genomic Positional Bias term is enabled
- CBN: whether the feed-forward module uses the CNN bottleneck (True) or GeGLU (False)

All models share the same core hyperparameters: a hidden dimension of 256, 6 encoder layers, 4 attention heads per layer, 0% dropout, batch size of 128, cosine-annealing learning-rate scheduler, and 40 warm-up epochs with no cool-down phase. Training uses a fixed missing ratio of 25% and learning rate 8×10^{-4} across all settings.

When the CNN bottleneck is used, we set the kernel size to 3 and the expansion factor to 2. Models are trained for 160 epochs on the 1KGP/EUR dataset and 70 epochs on the LOS/AA dataset to account for differences in sample size and convergence speed. Validation accuracy is reported as the primary metric for comparison. **Table S1** summarizes all experiment configurations and results of the validation set in this ablation study.

Table S1. Alation experiments for model components variation.

RGPB	CBN	Params (M)	FLOPs (G)	Size (MB)	Dataset	Accuracy
**T**	**T**	**6.32**	**1.44**	**24.10**	**1KGP**	**98.67%**

T	F	3.96	1.14	15.10	1KGP	98.37%
F	T	6.32	1.44	24.10	1KGP	98.10%
F	F	3.96	1.13	15.10	1KGP	96.52%
**T**	**T**	**6.32**	**1.441**	**24.10**	**LOS**	**98.67%**
T	F	3.96	1.139	15.10	LOS	98.12%
F	T	6.32	1.441	24.10	LOS	96.98%
F	F	3.96	1.139	15.10	LOS	96.66%

"1KGP" stands for 1KGP/EUR cohort, "LOS" stands for LOS/AA, "RGPG" as relative genomic positional bias, "CBN" as CNN bottleneck block, "T" as True, "F" as False. Experiment results achieved the highest imputation performance on the validation set of both datasets have been bolded.

The results reveal some insights:

1. RGPB and CNN Bottleneck each applied alone consistently improved the model performances across datasets.
2. The combination of RGPB + CNN Bottleneck provided the best performance on both datasets.

Alation II: Depth and hidden dimension scaling

Building on the findings from Ablation I, Ablation II further examines how model capacity affects performance by varying the hidden dimension and encoder depth, while toggling the RGPB and CNN bottleneck components. All other hyperparameters remain unchanged. Due to computational cost, this ablation is conducted only on the 1KGP/EUR dataset, with each model trained for 160 epochs. Validation accuracy is reported in **Table S2**.

Table S2. Alation experiments for depth and hidden dimension scaling.

Hidden_dim	Depth	Params (M)	FLOPs (G)	Size (MB)	Accuracy
128	2	0.53	0.13	2.02	95.42%
256	2	2.11	0.48	8.04	96.58%
512	2	8.41	1.85	32.09	97.77%
768	2	18.91	4.10	72.13	97.89%
768	4	37.80	8.20	144.21	98.26%
**768**	**6**	**56.70**	**12.29**	**216.29**	**99.01%**

Experiment results achieved the highest imputation performance on the validation set of the 1KGP dataset have been bolded.

From this ablation study, we observe a clear trend: increasing the hidden dimension and model depth consistently improves performance. However, this comes with a substantial increase in model size. For example, a configuration with a 768-dimensional hidden layer and 6 encoder

blocks (4 attention heads by default) contains approximately 57 million parameters, making it the largest model evaluated in our experiments.

Alation III: Effect of the Bottleneck Expansion Factor

Ablation III investigates how the shape of the convolutional bottleneck affects model performance. We evaluate a range of expansion factors from 0.5 to 2, while keeping all other settings aligned with the Ablation II configuration (768 hidden dimension, 6 encoder layers, 4 attention heads, RGPB enabled, and CBN with kernel size 3). Results for the European cohort of the 1KGP dataset are summarized in **Table S3**.

Table S3. Shapes of the bottleneck.

Bottleneck factor	Params (M)	FLOPs (G)	Size (MB)	Accuracy
0.5	24.84	6.08	94.76	98.16%
1	35.46	8.15	135.27	98.17%
**2**	**56.70**	**12.29**	**216.29**	**99.01%**

Experiment result achieved the highest imputation performance on the validation set of the 1KGP/EUR dataset have been bolded.

From this ablation, we observe that increasing the bottleneck expansion factor leads to steady improvements in model performance. However, the number of parameters grows almost linearly with the expansion factor, resulting in substantially higher training cost. Considering this trade-off between accuracy and computational efficiency, we chose not to further increase the model size.

Final architecture

Integrating the findings from all ablation studies, we arrive at the final configuration of the GenoBERT model. The complete architecture is summarized in **Table S4**.

Table S4. GenoBERT final architecture.

Context window	#Depth/#Heads	Hidden dimension	Weight Sharing	RGBP
130 (128+2)	6/4	768	None	Enabled
**Feed-forward**	**Bottleneck factor**	**Params (M)**	**FLOPs (G)**	**Size (MB)**
CNN Bottleneck	2.0	56.70	12.29	216.29

Weight initialization strategies

Effective weight initialization is critical to the stability and convergence of deep networks. Poor initialization can lead to vanishing or exploding gradients, degraded signal propagation, and unstable training dynamics. GenoBERT integrates diverse components—attention layers, the CNN

bottlenecks, and layer normalization components—each demanding tailored initialization to ensure stable learning.

At a high level, the goal of initialization is to maintain consistent variance across layers. If weights are initialized too small, signals shrink as they propagate through layers, leading to the vanishing gradient problem. Conversely, large initializations can cause gradients to explode, making optimization unstable.

In GenoBERT, all linear layers—including projection matrices $\boldsymbol{W_Q}$, $\boldsymbol{W_K}$, $\boldsymbol{W_V}$, and output projection head are initialized using Xavier Normal initialization[52]. For attention projection matrices $\boldsymbol{W_Q}, \boldsymbol{W_K}, \boldsymbol{W_V}$, GenoBERT empirically applies a fixed Xavier gain of $\sqrt{2}$, rather than the depth-dependent gain of $(8 * N)^{-1/4}$ proposed in the DeepNet framework for N-layer encoder-only Transformers. This adjustment reflects a practical consideration: GenoBERT's shallow architecture would otherwise result in overly large initialization variance when model depth N is small. Without sufficient regularization—such as dropout or warm-up—this could lead to gradient instability and activation explosion in early training.

In initializing the filters of the proposed CNN bottleneck layers, GenoBERT retains PyTorch's default Kaiming Uniform initialization[53,54]:

$$W \sim \mathcal{U}\left(-gain \cdot \sqrt{\frac{3}{\text{fan\_in}}}, gain \cdot \sqrt{\frac{3}{\text{fan\_in}}}\right) \quad (7)$$

With the default gain as $\sqrt{2}$, and fan_in $= C_{in} \times K$, suitable for ReLU's nonlinearity (C_{in} represents the number of channels, or the hidden dimension d to be more specific, and K represent the kernel size).

For initialize the scale parameters γ and the shift parameter β for the layer normalization, we use ones and zeros respectively.

The full initialization strategy for all different component of the GenoBERT model can be summarized as in **Table S5**.

Table S5. The summary of all initializations adopted in GenoBERT model.

Components	Initialization	Notes
ALL linear projections	Xavier Normal (gain varies): $W_{ij} \sim \mathcal{N}\left(0, \frac{gain^2}{n_{in}+n_{out}}\right)$	gain=$\sqrt{2}$; n_{in} and n_{out} are the input/output dimensions of the projection.
CNN Bottleneck filters	Kaiming Uniform: $W \sim \mathcal{U}\left(-gain \cdot \sqrt{\frac{3}{\text{fan\_in}}}, gain \cdot \sqrt{\frac{3}{\text{fan\_in}}}\right)$	gain=$\sqrt{2}$; fan_in = $d \times K$, d is the hidden dimension and K is the kernel size.
LayerNorm	Scale: γ ← 1, shift: β ← 0	

The detailed experiment settings for model training and performance evaluation can be found in the project's repository.

On choosing genotype segment length

To guide the selection of genotype segment length for GenoBERT, we examined variant spacing and LD structure in the datasets used in this study.

As shown in **Supplementary Fig. S1**, in the case of chromosome 22 of the 1KGP dataset, most consecutive SNP pairs lie within 1Kb, implying that a sequence of L $\approx$ 100–128 genotypes correspond to roughly 100Kb of physical distance. This provides an empirical upper bound for the genomic region represented by each model input window and supports the adequacy of the 128-SNP context length for local linkage modeling.

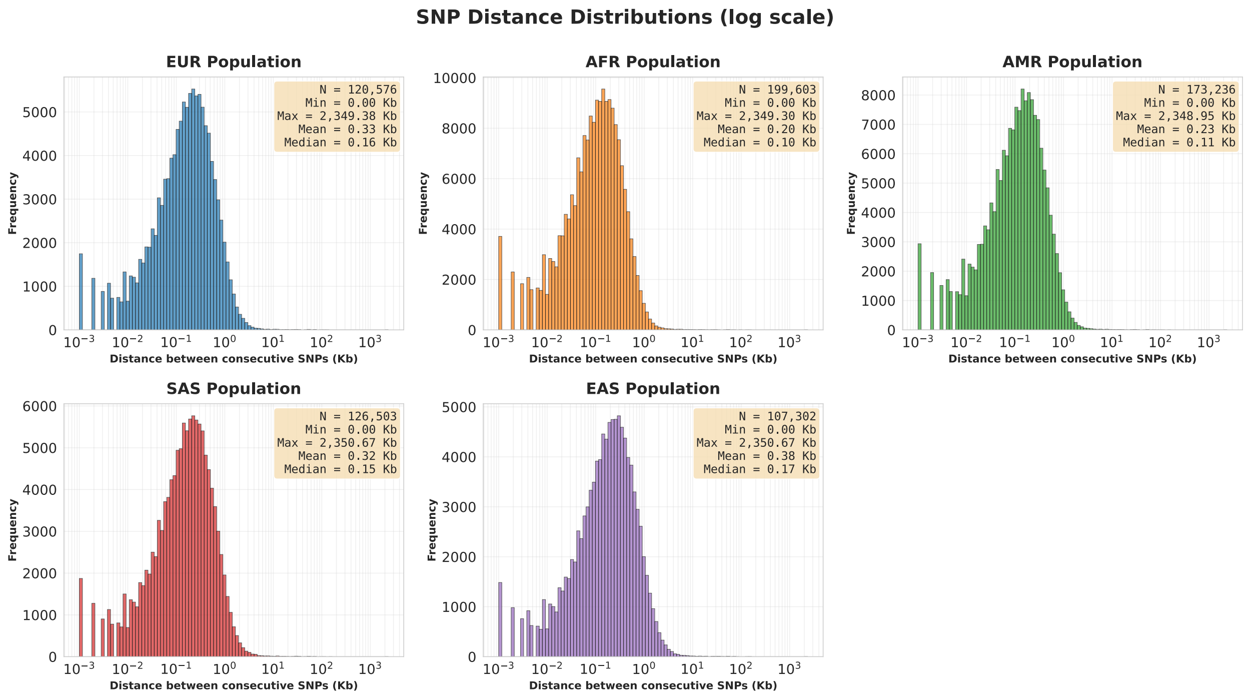

Figure S1 | Distribution of distances between consecutive SNPs in 1KGP populations. These log-scaled histograms illustrate adjacent-SNP distances on chromosome 22 for each of the five 1KGP populations. In all five groups, most consecutive SNPs fall within 1 Kb, defining the usual physical extent of short SNP sequences.

We further compared LD decay patterns between the 1KGP and LOS cohorts across matched ancestries (**Supplementary Fig. S2**). Consistent with established population-genetic patterns, European ancestry populations exhibit stronger and more extended LD than African ancestry populations. In both datasets and ancestries, LD decays rapidly with genomic distance, falling below $r^2 = 0.1$ within approximately 30 kb. These results indicate that a 128-SNP window provides sufficient genomic coverage to capture the majority of informative LD structure in both cohorts, despite differences in sample size and ancestry composition.

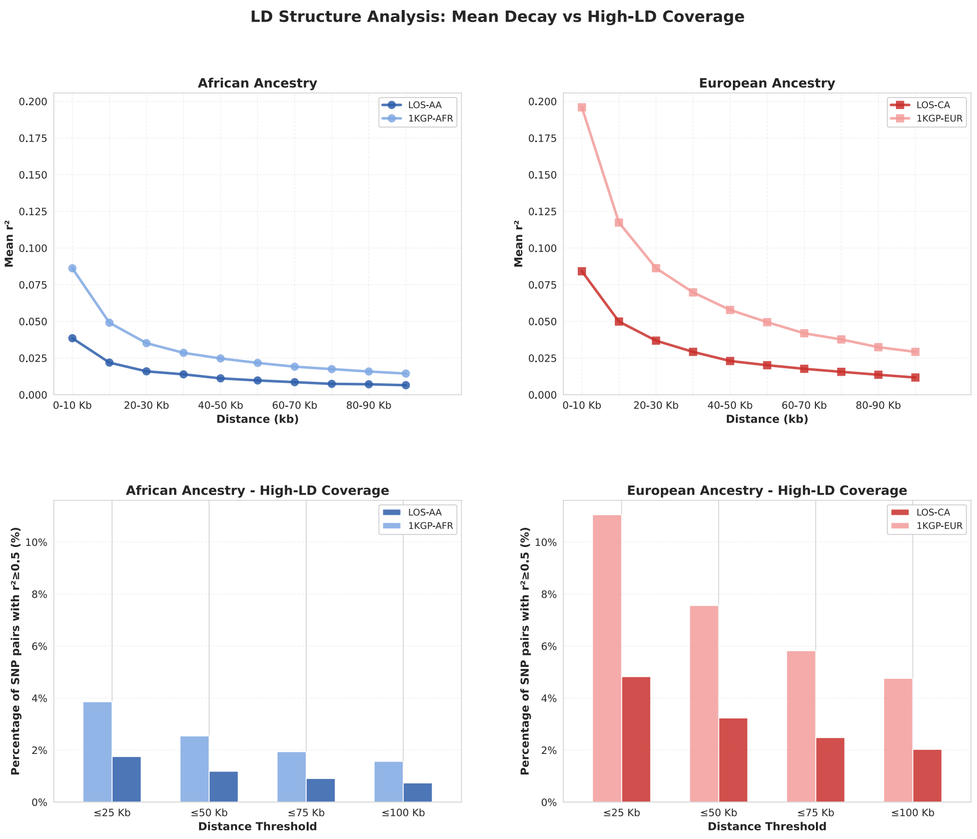

Figure S2 | Cross-dataset comparison of LD decay and high-LD coverage between 1KGP and LOS cohorts. Mean LD (r^2) decay curves (top) and the proportion of highly correlated SNP pairs ($r^2 \geq 0.5$) within distance thresholds (bottom) are shown for African and European ancestries. Within each ancestry, the 1KGP populations consistently display stronger and more extended LD than the corresponding LOS cohorts.

Comprehensive results

Tables S6–S35 summarize comprehensive performance results for all evaluated methods across LOS and 1KGP cohorts.

Models' performance on LOS/mixed populations cohort

Table S6. Beagle's performance on the mixed populations of LOS dataset

	MAF	#SNPs	Accuracy	r^2	Precision	Recall	F_1
**5% Missing**	**0.1%~0.5%**	**80535**	0.9969	0.8968	0.7493	0.7500	0.7497
	**0.5%~1%**	**43321**	0.9929	0.8978	0.7496	0.7490	0.7493
	**1%~10%**	**87563**	0.9707	0.9070	0.7500	0.7498	0.7499
	**10%~20%**	**17259**	0.8794	0.9053	0.7490	0.7489	0.7489
	**20%~30%**	**11414**	0.8183	0.8717	0.7489	0.7490	0.7489
	**30%~40%**	**9073**	0.7780	0.8412	0.7490	0.7491	0.7491
	**40%~50%**	**8187**	0.7591	0.8207	0.7493	0.7494	0.7494
	**Overall**	**257352**	0.9562	0.9191	0.7494	0.7494	0.7494
**15% Missing**	**MAF**	**#SNPs**	**Accuracy**	**r^2**	**Precision**	**Recall**	**F_1**
	**0.1%~0.5%**	**80535**	0.9968	0.8834	0.7469	0.7442	0.7455
	**0.5%~1%**	**43321**	0.9928	0.8902	0.7471	0.7455	0.7463
	**1%~10%**	**87563**	0.9703	0.9029	0.7487	0.7479	0.7483
	**10%~20%**	**17259**	0.8789	0.9030	0.7488	0.7487	0.7487
	**20%~30%**	**11414**	0.8171	0.8688	0.7476	0.7478	0.7477
	**30%~40%**	**9073**	0.7766	0.8379	0.7475	0.7478	0.7476
	**40%~50%**	**8187**	0.7576	0.8172	0.7476	0.7479	0.7478
	**Overall**	**257352**	0.9558	0.9166	0.7485	0.7482	0.7483
**25% Missing**	**MAF**	**#SNPs**	**Accuracy**	**r^2**	**Precision**	**Recall**	**F_1**
	**0.1%~0.5%**	**80535**	0.9967	0.8709	0.7452	0.7392	0.7422
	**0.5%~1%**	**43321**	0.9926	0.8813	0.7458	0.7423	0.7440
	**1%~10%**	**87563**	0.9697	0.8975	0.7463	0.7445	0.7454
	**10%~20%**	**17259**	0.8776	0.8997	0.7473	0.7469	0.7471
	**20%~30%**	**11414**	0.8158	0.8652	0.7462	0.7465	0.7463
	**30%~40%**	**9073**	0.7756	0.8342	0.7462	0.7469	0.7465
	**40%~50%**	**8187**	0.7567	0.8134	0.7463	0.7471	0.7467
	**Overall**	**257352**	0.9554	0.9135	0.7473	0.7466	0.7469
**50% Missing**	**MAF**	**#SNPs**	**Accuracy**	**r^2**	**Precision**	**Recall**	**F_1**
	**0.1%~0.5%**	**80535**	0.9962	0.8130	0.7392	0.7085	0.7228
	**0.5%~1%**	**43321**	0.9919	0.8433	0.7404	0.7231	0.7314
	**1%~10%**	**87563**	0.9679	0.8759	0.7414	0.7327	0.7369
	**10%~20%**	**17259**	0.8727	0.8850	0.7415	0.7391	0.7402
	**20%~30%**	**11414**	0.8096	0.8485	0.7385	0.7394	0.7389
	**30%~40%**	**9073**	0.7685	0.8156	0.7367	0.7395	0.7380
	**40%~50%**	**8187**	0.7494	0.7946	0.7362	0.7397	0.7379
	**Overall**	**257352**	0.9534	0.8995	0.7421	0.7380	0.7400

Table S7. SCDA's performance on the mixed populations of LOS dataset

	MAF	#SNPs	Accuracy	r^2	Precision	Recall	F_1
5% Missing	**0.1%~0.5%**	**80535**	0.9993	0.9354	0.9916	0.9411	0.9651
	**0.5%~1%**	**43321**	0.9988	0.9552	0.9914	0.9585	0.9744
	**1%~10%**	**87563**	0.9977	0.9817	0.9902	0.9852	0.9877
	**10%~20%**	**17259**	0.9943	0.9886	0.9904	0.9931	0.9917
	**20%~30%**	**11414**	0.9923	0.9860	0.9904	0.9929	0.9917
	**30%~40%**	**9073**	0.9915	0.9840	0.9909	0.9921	0.9915
	**40%~50%**	**8187**	0.9913	0.9828	0.9912	0.9915	0.9913
	**Overall**	**257352**	0.9975	0.9881	0.9909	0.9906	0.9907
15% Missing	**MAF**	**#SNPs**	**Accuracy**	**r^2**	**Precision**	**Recall**	**F_1**
	**0.1%~0.5%**	**80535**	0.9986	0.8809	0.9865	0.8945	0.9359
	**0.5%~1%**	**43321**	0.9977	0.9128	0.9866	0.9216	0.9519
	**1%~10%**	**87563**	0.9950	0.9589	0.9858	0.9637	0.9745
	**10%~20%**	**17259**	0.9898	0.9790	0.9863	0.9846	0.9855
	**20%~30%**	**11414**	0.9873	0.9762	0.9860	0.9864	0.9862
	**30%~40%**	**9073**	0.9861	0.9732	0.9858	0.9862	0.9860
	**40%~50%**	**8187**	0.9860	0.9714	0.9859	0.9860	0.9859
	**Overall**	**257352**	0.9953	0.9772	0.9873	0.9790	0.9831
25% Missing	**MAF**	**#SNPs**	**Accuracy**	**r^2**	**Precision**	**Recall**	**F_1**
	**0.1%~0.5%**	**80535**	0.9982	0.8394	0.9769	0.8637	0.9130
	**0.5%~1%**	**43321**	0.9964	0.8642	0.9792	0.8804	0.9244
	**1%~10%**	**87563**	0.9914	0.9283	0.9802	0.9350	0.9566
	**10%~20%**	**17259**	0.9824	0.9626	0.9805	0.9701	0.9752
	**20%~30%**	**11414**	0.9787	0.9587	0.9791	0.9750	0.9770
	**30%~40%**	**9073**	0.9769	0.9539	0.9776	0.9761	0.9768
	**40%~50%**	**8187**	0.9767	0.9509	0.9769	0.9766	0.9767
	**Overall**	**257352**	0.9922	0.9614	0.9826	0.9627	0.9724
50% Missing	**MAF**	**#SNPs**	**Accuracy**	**r^2**	**Precision**	**Recall**	**F_1**
	**0.1%~0.5%**	**80535**	0.9954	0.5603	0.8282	0.7201	0.7607
	**0.5%~1%**	**43321**	0.9911	0.6331	0.8847	0.7358	0.7931
	**1%~10%**	**87563**	0.9735	0.7552	0.9403	0.7921	0.8532
	**10%~20%**	**17259**	0.9232	0.8147	0.9420	0.8532	0.8916
	**20%~30%**	**11414**	0.9004	0.7828	0.9283	0.8694	0.8939
	**30%~40%**	**9073**	0.8885	0.7539	0.9132	0.8782	0.8904
	**40%~50%**	**8187**	0.8846	0.7348	0.9038	0.8823	0.8870
	**Overall**	**257352**	0.9709	0.8292	0.9517	0.8483	0.8944

Table S8. BiU-Net's performance on the mixed populations of LOS dataset

	MAF	#SNPs	Accuracy	r^2	Precision	Recall	F_1
**5% Missing**	**0.1%~0.5%**	**80535**	0.9999	0.9937	0.9987	0.9946	0.9966
	**0.5%~1%**	**43321**	0.9993	0.9721	0.9981	0.9741	0.9858
	**1%~10%**	**87563**	0.9983	0.9860	0.9960	0.9874	0.9917
	**10%~20%**	**17259**	0.9966	0.9931	0.9955	0.9948	0.9951
	**20%~30%**	**11414**	0.9962	0.9927	0.9957	0.9958	0.9957
	**30%~40%**	**9073**	0.9959	0.9921	0.9958	0.9959	0.9958
	**40%~50%**	**8187**	0.9959	0.9918	0.9959	0.9959	0.9959
	**Overall**	**257352**	0.9986	0.9932	0.9962	0.9937	0.9949
**15% Missing**	**MAF**	**#SNPs**	**Accuracy**	**r^2**	**Precision**	**Recall**	**F_1**
	**0.1%~0.5%**	**80535**	0.9991	0.9256	0.9968	0.9311	0.9617
	**0.5%~1%**	**43321**	0.9980	0.9259	0.9949	0.9312	0.9609
	**1%~10%**	**87563**	0.9949	0.9580	0.9883	0.9617	0.9746
	**10%~20%**	**17259**	0.9897	0.9787	0.9864	0.9839	0.9851
	**20%~30%**	**11414**	0.9881	0.9776	0.9867	0.9868	0.9868
	**30%~40%**	**9073**	0.9874	0.9757	0.9870	0.9873	0.9871
	**40%~50%**	**8187**	0.9875	0.9747	0.9874	0.9875	0.9874
	**Overall**	**257352**	0.9956	0.9787	0.9886	0.9796	0.9841
**25% Missing**	**MAF**	**#SNPs**	**Accuracy**	**r^2**	**Precision**	**Recall**	**F_1**
	**0.1%~0.5%**	**80535**	0.9986	0.8805	0.9952	0.8888	0.9358
	**0.5%~1%**	**43321**	0.9967	0.8788	0.9919	0.8866	0.9331
	**1%~10%**	**87563**	0.9914	0.9287	0.9808	0.9342	0.9563
	**10%~20%**	**17259**	0.9819	0.9628	0.9768	0.9712	0.9740
	**20%~30%**	**11414**	0.9790	0.9605	0.9769	0.9765	0.9767
	**30%~40%**	**9073**	0.9779	0.9573	0.9773	0.9776	0.9774
	**40%~50%**	**8187**	0.9779	0.9553	0.9778	0.9779	0.9778
	**Overall**	**257352**	0.9925	0.9634	0.9806	0.9647	0.9725
**50% Missing**	**MAF**	**#SNPs**	**Accuracy**	**r^2**	**Precision**	**Recall**	**F_1**
	**0.1%~0.5%**	**80535**	0.9968	0.7273	0.9923	0.7426	0.8262
	**0.5%~1%**	**43321**	0.9931	0.7436	0.9858	0.7559	0.8362
	**1%~10%**	**87563**	0.9803	0.8359	0.9615	0.8428	0.8934
	**10%~20%**	**17259**	0.9545	0.9046	0.9476	0.9222	0.9343
	**20%~30%**	**11414**	0.9459	0.8958	0.9440	0.9366	0.9402
	**30%~40%**	**9073**	0.9424	0.8861	0.9423	0.9407	0.9415
	**40%~50%**	**8187**	0.9424	0.8807	0.9423	0.9422	0.9423
	**Overall**	**257352**	0.9818	0.9101	0.9563	0.9106	0.9322

Table S9. STICI's performance on the mixed populations of LOS dataset

	MAF	#SNPs	Accuracy	r^2	Precision	Recall	F_1
5% Missing	**0.1%~0.5%**	**80535**	0.9969	0.7037	0.9180	0.7721	0.8298
	**0.5%~1%**	**43321**	0.9950	0.7938	0.9424	0.8309	0.8790
	**1%~10%**	**87563**	0.9896	0.9000	0.9651	0.9153	0.9389
	**10%~20%**	**17259**	0.9758	0.9374	0.9707	0.9562	0.9633
	**20%~30%**	**11414**	0.9701	0.9305	0.9699	0.9621	0.9659
	**30%~40%**	**9073**	0.9678	0.9246	0.9686	0.9654	0.9669
	**40%~50%**	**8187**	0.9674	0.9202	0.9680	0.9669	0.9673
	**Overall**	**257352**	0.9895	0.9394	0.9722	0.9460	0.9587
15% Missing	**MAF**	**#SNPs**	**Accuracy**	**r^2**	**Precision**	**Recall**	**F_1**
	**0.1%~0.5%**	**80535**	0.9974	0.7602	0.9530	0.7995	0.8613
	**0.5%~1%**	**43321**	0.9959	0.8385	0.9644	0.8608	0.9064
	**1%~10%**	**87563**	0.9912	0.9196	0.9748	0.9298	0.9513
	**10%~20%**	**17259**	0.9802	0.9525	0.9770	0.9653	0.9711
	**20%~30%**	**11414**	0.9762	0.9482	0.9764	0.9709	0.9736
	**30%~40%**	**9073**	0.9742	0.9426	0.9751	0.9728	0.9739
	**40%~50%**	**8187**	0.9741	0.9391	0.9745	0.9738	0.9741
	**Overall**	**257352**	0.9914	0.9529	0.9792	0.9565	0.9676
25% Missing	**MAF**	**#SNPs**	**Accuracy**	**r^2**	**Precision**	**Recall**	**F_1**
	**0.1%~0.5%**	**80535**	0.9975	0.7757	0.9658	0.8057	0.8702
	**0.5%~1%**	**43321**	0.9958	0.8382	0.9713	0.8569	0.9068
	**1%~10%**	**87563**	0.9905	0.9163	0.9763	0.9253	0.9495
	**10%~20%**	**17259**	0.9792	0.9526	0.9767	0.9643	0.9704
	**20%~30%**	**11414**	0.9754	0.9487	0.9758	0.9704	0.9731
	**30%~40%**	**9073**	0.9736	0.9433	0.9744	0.9724	0.9734
	**40%~50%**	**8187**	0.9735	0.9401	0.9738	0.9733	0.9735
	**Overall**	**257352**	0.9910	0.9527	0.9795	0.9554	0.9672
50% Missing	**MAF**	**#SNPs**	**Accuracy**	**r^2**	**Precision**	**Recall**	**F_1**
	**0.1%~0.5%**	**80535**	0.9967	0.7139	0.9750	0.7377	0.8186
	**0.5%~1%**	**43321**	0.9940	0.7764	0.9718	0.7951	0.8640
	**1%~10%**	**87563**	0.9865	0.8857	0.9700	0.8955	0.9296
	**10%~20%**	**17259**	0.9723	0.9403	0.9684	0.9538	0.9609
	**20%~30%**	**11414**	0.9673	0.9354	0.9665	0.9620	0.9642
	**30%~40%**	**9073**	0.9646	0.9282	0.9647	0.9636	0.9641
	**40%~50%**	**8187**	0.9647	0.9251	0.9648	0.9646	0.9647
	**Overall**	**257352**	0.9877	0.9383	0.9718	0.9401	0.9554

Table S10. GenoBERT's performance on the mixed populations of LOS dataset

	MAF	#SNPs	Accuracy	r^2	Precision	Recall	F_1
5% Missing	**0.1%~0.5%**	**80535**	0.9999	0.9933	0.9987	0.9958	0.9972
	**0.5%~1%**	**43321**	0.9996	0.9825	0.9982	0.9851	0.9916
	**1%~10%**	**87563**	0.9992	0.9929	0.9982	0.9943	0.9962
	**10%~20%**	**17259**	0.9986	0.9968	0.9983	0.9979	0.9981
	**20%~30%**	**11414**	0.9985	0.9968	0.9984	0.9983	0.9983
	**30%~40%**	**9073**	0.9984	0.9965	0.9983	0.9983	0.9983
	**40%~50%**	**8187**	0.9984	0.9964	0.9984	0.9984	0.9984
	**Overall**	**257352**	0.9994	0.9966	0.9985	0.9972	0.9978
15% Missing	**MAF**	**#SNPs**	**Accuracy**	**r^2**	**Precision**	**Recall**	**F_1**
	**0.1%~0.5%**	**80535**	0.9993	0.9322	0.9949	0.9432	0.9677
	**0.5%~1%**	**43321**	0.9987	0.9491	0.9943	0.9576	0.9753
	**1%~10%**	**87563**	0.9975	0.9773	0.9943	0.9816	0.9879
	**10%~20%**	**17259**	0.9954	0.9892	0.9946	0.9928	0.9937
	**20%~30%**	**11414**	0.9949	0.9893	0.9947	0.9943	0.9945
	**30%~40%**	**9073**	0.9946	0.9884	0.9946	0.9945	0.9946
	**40%~50%**	**8187**	0.9946	0.9878	0.9946	0.9946	0.9946
	**Overall**	**257352**	0.9978	0.9883	0.9952	0.9898	0.9925
25% Missing	**MAF**	**#SNPs**	**Accuracy**	**r^2**	**Precision**	**Recall**	**F_1**
	**0.1%~0.5%**	**80535**	0.9988	0.8846	0.9899	0.9049	0.9437
	**0.5%~1%**	**43321**	0.9978	0.9104	0.9897	0.9258	0.9558
	**1%~10%**	**87563**	0.9954	0.9578	0.9902	0.9655	0.9776
	**10%~20%**	**17259**	0.9910	0.9786	0.9903	0.9854	0.9878
	**20%~30%**	**11414**	0.9900	0.9784	0.9900	0.9885	0.9893
	**30%~40%**	**9073**	0.9894	0.9767	0.9896	0.9892	0.9894
	**40%~50%**	**8187**	0.9894	0.9752	0.9895	0.9894	0.9894
	**Overall**	**257352**	0.9959	0.9778	0.9914	0.9807	0.9860
50% Missing	**MAF**	**#SNPs**	**Accuracy**	**r^2**	**Precision**	**Recall**	**F_1**
	**0.1%~0.5%**	**80535**	0.9968	0.6749	0.9596	0.7444	0.8252
	**0.5%~1%**	**43321**	0.9939	0.7322	0.9670	0.7873	0.8610
	**1%~10%**	**87563**	0.9842	0.8437	0.9747	0.8718	0.9182
	**10%~20%**	**17259**	0.9601	0.8944	0.9693	0.9262	0.9465
	**20%~30%**	**11414**	0.9512	0.8821	0.9617	0.9382	0.9491
	**30%~40%**	**9073**	0.9468	0.8694	0.9543	0.9430	0.9478
	**40%~50%**	**8187**	0.9456	0.8598	0.9497	0.9449	0.9464
	**Overall**	**257352**	0.9842	0.9029	0.9758	0.9177	0.9452

Models' performance on LOS/AA cohort

Table S11. Beagle's performance on the African American cohort of LOS dataset

	MAF	#SNPs	Accuracy	r^2	Precision	Recall	F_1
5% Missing	0.1%~0.5%	72297	0.9966	0.9123	0.7516	0.7527	0.7522
	0.5%~1%	41655	0.9928	0.9173	0.7509	0.7517	0.7513
	1%~10%	120279	0.9653	0.9059	0.7504	0.7504	0.7504
	10%~20%	30423	0.8802	0.8956	0.7503	0.7503	0.7503
	20%~30%	17490	0.8194	0.8700	0.7497	0.7497	0.7497
	30%~40%	13326	0.7797	0.8418	0.7501	0.7502	0.7501
	40%~50%	12129	0.7591	0.8201	0.7498	0.7499	0.7498
	Overall	307599	0.9435	0.9138	0.7503	0.7503	0.7503
15% Missing	MAF	#SNPs	Accuracy	r^2	Precision	Recall	F_1
	0.1%~0.5%	72297	0.9965	0.9032	0.7459	0.7465	0.7462
	0.5%~1%	41655	0.9925	0.9078	0.7462	0.7451	0.7457
	1%~10%	120279	0.9646	0.9012	0.7474	0.7472	0.7473
	10%~20%	30423	0.8788	0.8923	0.7481	0.7482	0.7481
	20%~30%	17490	0.8175	0.8663	0.7475	0.7476	0.7476
	30%~40%	13326	0.7779	0.8380	0.7481	0.7484	0.7482
	40%~50%	12129	0.7569	0.8156	0.7472	0.7477	0.7475
	Overall	307599	0.9428	0.9107	0.7481	0.7480	0.7481
25% Missing	MAF	#SNPs	Accuracy	r^2	Precision	Recall	F_1
	0.1%~0.5%	72297	0.9963	0.8909	0.7418	0.7395	0.7406
	0.5%~1%	41655	0.9923	0.8988	0.7422	0.7394	0.7408
	1%~10%	120279	0.9638	0.8959	0.7436	0.7430	0.7433
	10%~20%	30423	0.8765	0.8880	0.7443	0.7443	0.7443
	20%~30%	17490	0.8147	0.8616	0.7439	0.7443	0.7441
	30%~40%	13326	0.7744	0.8327	0.7441	0.7447	0.7444
	40%~50%	12129	0.7532	0.8097	0.7430	0.7439	0.7434
	Overall	307599	0.9417	0.9069	0.7446	0.7444	0.7445
50% Missing	MAF	#SNPs	Accuracy	r^2	Precision	Recall	F_1
	0.1%~0.5%	72297	0.9959	0.8434	0.7403	0.7147	0.7267
	0.5%~1%	41655	0.9916	0.8686	0.7399	0.7253	0.7323
	1%~10%	120279	0.9624	0.8794	0.7416	0.7374	0.7394
	10%~20%	30423	0.8739	0.8757	0.7422	0.7414	0.7418
	20%~30%	17490	0.8105	0.8471	0.7391	0.7401	0.7396
	30%~40%	13326	0.7688	0.8164	0.7370	0.7392	0.7381
	40%~50%	12129	0.7485	0.7936	0.7362	0.7393	0.7377
	Overall	307599	0.9400	0.8951	0.7421	0.7401	0.7410

Table S12. SCDA's performance on the African American cohort of LOS dataset

	MAF	#SNPs	Accuracy	r^2	Precision	Recall	F_1
**5% Missing**	**0.1%~0.5%**	**72297**	0.9944	0.5640	0.7086	0.9448	0.8016
	**0.5%~1%**	**41655**	0.9932	0.7058	0.8099	0.9366	0.8667
	**1%~10%**	**120279**	0.9850	0.8485	0.9233	0.9158	0.9195
	**10%~20%**	**30423**	0.9584	0.8691	0.9491	0.9172	0.9326
	**20%~30%**	**17490**	0.9405	0.8447	0.9457	0.9194	0.9316
	**30%~40%**	**13326**	0.9296	0.8169	0.9385	0.9221	0.9287
	**40%~50%**	**12129**	0.9241	0.7951	0.9311	0.9229	0.9247
	**Overall**	**307599**	0.9784	0.8730	0.9442	0.9198	0.9317
**15% Missing**	**MAF**	**#SNPs**	**Accuracy**	**r^2**	**Precision**	**Recall**	**F_1**
	**0.1%~0.5%**	**72297**	0.9919	0.4260	0.6420	0.8997	0.7374
	**0.5%~1%**	**41655**	0.9891	0.5497	0.7522	0.8587	0.7998
	**1%~10%**	**120279**	0.9757	0.7383	0.9074	0.8570	0.8810
	**10%~20%**	**30423**	0.9306	0.7602	0.9398	0.8605	0.8963
	**20%~30%**	**17490**	0.8985	0.7183	0.9288	0.8619	0.8900
	**30%~40%**	**13326**	0.8782	0.6753	0.9126	0.8647	0.8809
	**40%~50%**	**12129**	0.8680	0.6451	0.8997	0.8656	0.8723
	**Overall**	**307599**	0.9640	0.7702	0.9347	0.8620	0.8960
**25% Missing**	**MAF**	**#SNPs**	**Accuracy**	**r^2**	**Precision**	**Recall**	**F_1**
	**0.1%~0.5%**	**72297**	0.9902	0.3369	0.6033	0.8422	0.6906
	**0.5%~1%**	**41655**	0.9863	0.4464	0.7161	0.7946	0.7515
	**1%~10%**	**120279**	0.9670	0.6373	0.8953	0.7951	0.8405
	**10%~20%**	**30423**	0.9016	0.6551	0.9315	0.8000	0.8551
	**20%~30%**	**17490**	0.8540	0.6020	0.9143	0.8006	0.8433
	**30%~40%**	**13326**	0.8233	0.5518	0.8925	0.8034	0.8291
	**40%~50%**	**12129**	0.8080	0.5188	0.8771	0.8043	0.8172
	**Overall**	**307599**	0.9497	0.6713	0.9270	0.8008	0.8564
**50% Missing**	**MAF**	**#SNPs**	**Accuracy**	**r^2**	**Precision**	**Recall**	**F_1**
	**0.1%~0.5%**	**72297**	0.9892	0.1980	0.5696	0.6551	0.6052
	**0.5%~1%**	**41655**	0.9830	0.2733	0.6853	0.6222	0.6502
	**1%~10%**	**120279**	0.9467	0.4118	0.8845	0.6244	0.7184
	**10%~20%**	**30423**	0.8225	0.4085	0.9156	0.6303	0.7188
	**20%~30%**	**17490**	0.7309	0.3508	0.8873	0.6295	0.6943
	**30%~40%**	**13326**	0.6699	0.3041	0.8592	0.6316	0.6705
	**40%~50%**	**12129**	0.6397	0.2773	0.8423	0.6326	0.6539
	**Overall**	**307599**	0.9130	0.4350	0.9179	0.6296	0.7289

Table S13. BiU-Net's performance on the African American cohort of LOS dataset

	MAF	#SNPs	Accuracy	r^2	Precision	Recall	F_1
5% Missing	**0.1%~0.5%**	**72297**	1.0000	0.9976	0.9975	0.9989	0.9982
	**0.5%~1%**	**41655**	0.9999	0.9956	0.9982	0.9959	0.9971
	**1%~10%**	**120279**	0.9970	0.9788	0.9963	0.9792	0.9876
	**10%~20%**	**30423**	0.9939	0.9872	0.9933	0.9884	0.9908
	**20%~30%**	**17490**	0.9928	0.9882	0.9925	0.9909	0.9917
	**30%~40%**	**13326**	0.9927	0.9882	0.9926	0.9922	0.9924
	**40%~50%**	**12129**	0.9928	0.9880	0.9928	0.9927	0.9927
	**Overall**	**307599**	0.9972	0.9896	0.9945	0.9887	0.9916
15% Missing	**MAF**	**#SNPs**	**Accuracy**	**r^2**	**Precision**	**Recall**	**F_1**
	**0.1%~0.5%**	**72297**	0.9994	0.9587	0.9897	0.9644	0.9767
	**0.5%~1%**	**41655**	0.9978	0.9316	0.9923	0.9260	0.9568
	**1%~10%**	**120279**	0.9906	0.9333	0.9875	0.9355	0.9600
	**10%~20%**	**30423**	0.9792	0.9549	0.9788	0.9602	0.9692
	**20%~30%**	**17490**	0.9752	0.9571	0.9754	0.9683	0.9718
	**30%~40%**	**13326**	0.9748	0.9571	0.9751	0.9729	0.9739
	**40%~50%**	**12129**	0.9752	0.9564	0.9752	0.9748	0.9750
	**Overall**	**307599**	0.9903	0.9632	0.9825	0.9611	0.9715
25% Missing	**MAF**	**#SNPs**	**Accuracy**	**r^2**	**Precision**	**Recall**	**F_1**
	**0.1%~0.5%**	**72297**	0.9986	0.9009	0.9705	0.9199	0.9437
	**0.5%~1%**	**41655**	0.9960	0.8776	0.9789	0.8740	0.9201
	**1%~10%**	**120279**	0.9835	0.8813	0.9752	0.8885	0.9274
	**10%~20%**	**30423**	0.9600	0.9100	0.9614	0.9232	0.9411
	**20%~30%**	**17490**	0.9509	0.9106	0.9540	0.9367	0.9448
	**30%~40%**	**13326**	0.9495	0.9087	0.9517	0.9454	0.9482
	**40%~50%**	**12129**	0.9499	0.9058	0.9506	0.9490	0.9495
	**Overall**	**307599**	0.9818	0.9276	0.9678	0.9266	0.9462
50% Missing	**MAF**	**#SNPs**	**Accuracy**	**r^2**	**Precision**	**Recall**	**F_1**
	**0.1%~0.5%**	**72297**	0.9946	0.5963	0.7870	0.7701	0.7747
	**0.5%~1%**	**41655**	0.9898	0.6583	0.8495	0.7366	0.7804
	**1%~10%**	**120279**	0.9605	0.6949	0.9035	0.7474	0.8079
	**10%~20%**	**30423**	0.8875	0.7211	0.8958	0.7825	0.8279
	**20%~30%**	**17490**	0.8476	0.6902	0.8747	0.8000	0.8286
	**30%~40%**	**13326**	0.8309	0.6577	0.8595	0.8151	0.8291
	**40%~50%**	**12129**	0.8255	0.6344	0.8490	0.8221	0.8268
	**Overall**	**307599**	0.9479	0.7597	0.9081	0.7925	0.8417

Table S14. STICI's performance on the African American cohort of LOS dataset

	MAF	#SNPs	Accuracy	r^2	Precision	Recall	F_1
**5% Missing**	**0.1%~0.5%**	**72297**	0.9960	0.7324	0.9346	0.7184	0.7925
	**0.5%~1%**	**41655**	0.9943	0.8252	0.9474	0.8119	0.8680
	**1%~10%**	**120279**	0.9881	0.9047	0.9637	0.9173	0.9394
	**10%~20%**	**30423**	0.9763	0.9320	0.9683	0.9548	0.9614
	**20%~30%**	**17490**	0.9684	0.9230	0.9662	0.9593	0.9627
	**30%~40%**	**13326**	0.9651	0.9142	0.9653	0.9623	0.9638
	**40%~50%**	**12129**	0.9638	0.9074	0.9643	0.9633	0.9636
	**Overall**	**307599**	0.9866	0.9358	0.9693	0.9451	0.9569
**15% Missing**	**MAF**	**#SNPs**	**Accuracy**	**r^2**	**Precision**	**Recall**	**F_1**
	**0.1%~0.5%**	**72297**	0.9966	0.7701	0.9503	0.7556	0.8269
	**0.5%~1%**	**41655**	0.9946	0.8344	0.9567	0.8213	0.8777
	**1%~10%**	**120279**	0.9886	0.9116	0.9687	0.9240	0.9453
	**10%~20%**	**30423**	0.9775	0.9392	0.9715	0.9591	0.9652
	**20%~30%**	**17490**	0.9706	0.9324	0.9693	0.9635	0.9664
	**30%~40%**	**13326**	0.9682	0.9262	0.9687	0.9664	0.9675
	**40%~50%**	**12129**	0.9673	0.9208	0.9677	0.9670	0.9673
	**Overall**	**307599**	0.9874	0.9427	0.9730	0.9508	0.9616
**25% Missing**	**MAF**	**#SNPs**	**Accuracy**	**r^2**	**Precision**	**Recall**	**F_1**
	**0.1%~0.5%**	**72297**	0.9967	0.7812	0.9585	0.7653	0.8368
	**0.5%~1%**	**41655**	0.9944	0.8301	0.9606	0.8152	0.8747
	**1%~10%**	**120279**	0.9872	0.9037	0.9689	0.9165	0.9412
	**10%~20%**	**30423**	0.9749	0.9354	0.9698	0.9555	0.9625
	**20%~30%**	**17490**	0.9679	0.9299	0.9671	0.9609	0.9640
	**30%~40%**	**13326**	0.9657	0.9243	0.9662	0.9640	0.9651
	**40%~50%**	**12129**	0.9650	0.9193	0.9653	0.9647	0.9650
	**Overall**	**307599**	0.9863	0.9397	0.9719	0.9475	0.9594
**50% Missing**	**MAF**	**#SNPs**	**Accuracy**	**r^2**	**Precision**	**Recall**	**F_1**
	**0.1%~0.5%**	**72297**	0.9961	0.7451	0.9701	0.7218	0.8026
	**0.5%~1%**	**41655**	0.9927	0.7825	0.9637	0.7571	0.8316
	**1%~10%**	**120279**	0.9814	0.8664	0.9611	0.8805	0.9171
	**10%~20%**	**30423**	0.9641	0.9139	0.9578	0.9381	0.9477
	**20%~30%**	**17490**	0.9548	0.9089	0.9532	0.9466	0.9498
	**30%~40%**	**13326**	0.9519	0.9024	0.9519	0.9504	0.9511
	**40%~50%**	**12129**	0.9520	0.8983	0.9520	0.9518	0.9519
	**Overall**	**307599**	0.9807	0.9202	0.9612	0.9283	0.9441

Table S15. GenoBERT's performance on the African American cohort of LOS dataset

	MAF	#SNPs	Accuracy	r^2	Precision	Recall	F_1
5% Missing	0.1%~0.5%	72297	1.0000	0.9979	0.9995	0.9988	0.9992
	0.5%~1%	41655	0.9999	0.9963	0.9990	0.9974	0.9982
	1%~10%	120279	0.9991	0.9933	0.9982	0.9944	0.9963
	10%~20%	30423	0.9986	0.9965	0.9983	0.9977	0.9980
	20%~30%	17490	0.9982	0.9963	0.9982	0.9979	0.9981
	30%~40%	13326	0.9981	0.9961	0.9981	0.9981	0.9981
	40%~50%	12129	0.9982	0.9960	0.9982	0.9982	0.9982
	Overall	307599	0.9992	0.9967	0.9985	0.9973	0.9979
15% Missing	MAF	#SNPs	Accuracy	r^2	Precision	Recall	F_1
	0.1%~0.5%	72297	0.9996	0.9676	0.9977	0.9687	0.9828
	0.5%~1%	41655	0.9985	0.9518	0.9958	0.9494	0.9715
	1%~10%	120279	0.9972	0.9790	0.9943	0.9828	0.9885
	10%~20%	30423	0.9956	0.9893	0.9947	0.9930	0.9938
	20%~30%	17490	0.9945	0.9885	0.9943	0.9937	0.9940
	30%~40%	13326	0.9942	0.9879	0.9942	0.9941	0.9942
	40%~50%	12129	0.9943	0.9874	0.9943	0.9943	0.9943
	Overall	307599	0.9974	0.9888	0.9950	0.9906	0.9928
25% Missing	MAF	#SNPs	Accuracy	r^2	Precision	Recall	F_1
	0.1%~0.5%	72297	0.9990	0.9263	0.9939	0.9285	0.9590
	0.5%~1%	41655	0.9973	0.9154	0.9911	0.9121	0.9483
	1%~10%	120279	0.9949	0.9620	0.9893	0.9693	0.9791
	10%~20%	30423	0.9919	0.9797	0.9901	0.9869	0.9885
	20%~30%	17490	0.9897	0.9785	0.9893	0.9882	0.9887
	30%~40%	13326	0.9892	0.9772	0.9893	0.9890	0.9891
	40%~50%	12129	0.9893	0.9765	0.9893	0.9893	0.9893
	Overall	307599	0.9951	0.9791	0.9907	0.9827	0.9867
50% Missing	MAF	#SNPs	Accuracy	r^2	Precision	Recall	F_1
	0.1%~0.5%	72297	0.9964	0.7075	0.9288	0.7819	0.8383
	0.5%~1%	41655	0.9930	0.7565	0.9452	0.7876	0.8497
	1%~10%	120279	0.9829	0.8621	0.9658	0.8922	0.9260
	10%~20%	30423	0.9662	0.9076	0.9666	0.9397	0.9527
	20%~30%	17490	0.9560	0.8987	0.9605	0.9460	0.9529
	30%~40%	13326	0.9532	0.8903	0.9567	0.9507	0.9535
	40%~50%	12129	0.9529	0.8854	0.9544	0.9525	0.9532
	Overall	307599	0.9818	0.9133	0.9689	0.9318	0.9496

Models' performance on LOS/CA cohort

Table S16. Beagle's performance on the Caucasian cohort of LOS dataset

	MAF	#SNPs	Accuracy	r^2	Precision	Recall	F_1
**5% Missing**	**0.1%~0.5%**	**52524**	0.9973	0.9284	0.7477	0.7491	0.7484
	**0.5%~1%**	**20151**	0.9933	0.9323	0.7456	0.7461	0.7458
	**1%~10%**	**54011**	0.9623	0.9178	0.7498	0.7495	0.7497
	**10%~20%**	**19838**	0.8751	0.8992	0.7496	0.7496	0.7496
	**20%~30%**	**15717**	0.8144	0.8686	0.7494	0.7494	0.7494
	**30%~40%**	**13401**	0.7738	0.8367	0.7488	0.7489	0.7488
	**40%~50%**	**12757**	0.7537	0.8144	0.7486	0.7487	0.7486
	**Overall**	**188399**	0.9263	0.9138	0.7493	0.7492	0.7493
**15% Missing**	**MAF**	**#SNPs**	**Accuracy**	**r^2**	**Precision**	**Recall**	**F_1**
	**0.1%~0.5%**	**52524**	0.9972	0.9181	0.7447	0.7435	0.7441
	**0.5%~1%**	**20151**	0.9931	0.9235	0.7477	0.7440	0.7458
	**1%~10%**	**54011**	0.9617	0.9136	0.7486	0.7474	0.7480
	**10%~20%**	**19838**	0.8749	0.8970	0.7501	0.7499	0.7500
	**20%~30%**	**15717**	0.8141	0.8665	0.7492	0.7493	0.7493
	**30%~40%**	**13401**	0.7737	0.8345	0.7487	0.7490	0.7489
	**40%~50%**	**12757**	0.7542	0.8126	0.7489	0.7492	0.7490
	**Overall**	**188399**	0.9261	0.9118	0.7495	0.7493	0.7494
**25% Missing**	**MAF**	**#SNPs**	**Accuracy**	**r^2**	**Precision**	**Recall**	**F_1**
	**0.1%~0.5%**	**52524**	0.9971	0.9074	0.7452	0.7396	0.7424
	**0.5%~1%**	**20151**	0.9929	0.9141	0.7443	0.7376	0.7409
	**1%~10%**	**54011**	0.9614	0.9094	0.7491	0.7467	0.7479
	**10%~20%**	**19838**	0.8736	0.8938	0.7485	0.7482	0.7484
	**20%~30%**	**15717**	0.8126	0.8631	0.7476	0.7478	0.7477
	**30%~40%**	**13401**	0.7721	0.8308	0.7469	0.7474	0.7471
	**40%~50%**	**12757**	0.7524	0.8086	0.7467	0.7474	0.7470
	**Overall**	**188399**	0.9254	0.9091	0.7484	0.7479	0.7482
**50% Missing**	**MAF**	**#SNPs**	**Accuracy**	**r^2**	**Precision**	**Recall**	**F_1**
	**0.1%~0.5%**	**52524**	0.9966	0.8559	0.7368	0.6999	0.7167
	**0.5%~1%**	**20151**	0.9918	0.8752	0.7359	0.7060	0.7199
	**1%~10%**	**54011**	0.9583	0.8884	0.7396	0.7301	0.7347
	**10%~20%**	**19838**	0.8673	0.8795	0.7391	0.7378	0.7384
	**20%~30%**	**15717**	0.8047	0.8482	0.7376	0.7384	0.7380
	**30%~40%**	**13401**	0.7633	0.8143	0.7359	0.7381	0.7370
	**40%~50%**	**12757**	0.7424	0.7906	0.7344	0.7373	0.7358
	**Overall**	**188399**	0.9216	0.8964	0.7396	0.7374	0.7385

Table S17. SCDA's performance on the Caucasian cohort of LOS dataset

	MAF	#SNPs	Accuracy	r^2	Precision	Recall	F_1
5% Missing	**0.1%~0.5%**	**52524**	0.9858	0.3219	0.5053	0.9166	0.6180
	**0.5%~1%**	**20151**	0.9844	0.5384	0.6652	0.9138	0.7601
	**1%~10%**	**54011**	0.9745	0.7977	0.8776	0.8994	0.8883
	**10%~20%**	**19838**	0.9453	0.8463	0.9277	0.9035	0.9152
	**20%~30%**	**15717**	0.9314	0.8334	0.9336	0.9124	0.9223
	**30%~40%**	**13401**	0.9229	0.8095	0.9293	0.9166	0.9216
	**40%~50%**	**12757**	0.9216	0.7970	0.9269	0.9205	0.9218
	**Overall**	**188399**	0.9648	0.8455	0.9213	0.9113	0.9162
15% Missing	**MAF**	**#SNPs**	**Accuracy**	**r^2**	**Precision**	**Recall**	**F_1**
	**0.1%~0.5%**	**52524**	0.9813	0.2320	0.4558	0.8720	0.5577
	**0.5%~1%**	**20151**	0.9787	0.4109	0.6050	0.8467	0.6944
	**1%~10%**	**54011**	0.9640	0.6952	0.8552	0.8497	0.8524
	**10%~20%**	**19838**	0.9188	0.7460	0.9163	0.8547	0.8829
	**20%~30%**	**15717**	0.8929	0.7176	0.9156	0.8621	0.8847
	**30%~40%**	**13401**	0.8771	0.6816	0.9037	0.8667	0.8789
	**40%~50%**	**12757**	0.8721	0.6614	0.8967	0.8702	0.8753
	**Overall**	**188399**	0.9473	0.7486	0.9076	0.8612	0.8833
25% Missing	**MAF**	**#SNPs**	**Accuracy**	**r^2**	**Precision**	**Recall**	**F_1**
	**0.1%~0.5%**	**52524**	0.9784	0.1780	0.4274	0.8244	0.5193
	**0.5%~1%**	**20151**	0.9750	0.3274	0.5686	0.7962	0.6516
	**1%~10%**	**54011**	0.9542	0.6009	0.8370	0.7961	0.8156
	**10%~20%**	**19838**	0.8904	0.6467	0.9057	0.8008	0.8456
	**20%~30%**	**15717**	0.8510	0.6074	0.8996	0.8068	0.8421
	**30%~40%**	**13401**	0.8270	0.5652	0.8828	0.8120	0.8320
	**40%~50%**	**12757**	0.8177	0.5407	0.8731	0.8148	0.8250
	**Overall**	**188399**	0.9296	0.6537	0.8955	0.8066	0.8469
50% Missing	**MAF**	**#SNPs**	**Accuracy**	**r^2**	**Precision**	**Recall**	**F_1**
	**0.1%~0.5%**	**52524**	0.9783	0.0965	0.3948	0.6481	0.4610
	**0.5%~1%**	**20151**	0.9721	0.1842	0.5218	0.6240	0.5646
	**1%~10%**	**54011**	0.9320	0.3842	0.8127	0.6348	0.7052
	**10%~20%**	**19838**	0.8096	0.4033	0.8857	0.6400	0.7191
	**20%~30%**	**15717**	0.7286	0.3563	0.8688	0.6429	0.7012
	**30%~40%**	**13401**	0.6774	0.3160	0.8462	0.6478	0.6819
	**40%~50%**	**12757**	0.6550	0.2937	0.8345	0.6492	0.6695
	**Overall**	**188399**	0.8825	0.4200	0.8759	0.6430	0.7272

Table S18. BiU-Net's performance on the Caucasian cohort of LOS dataset

	MAF	#SNPs	Accuracy	r^2	Precision	Recall	F_1
5% Missing	0.1%~0.5%	52524	1.0000	0.9970	0.9983	0.9972	0.9977
	0.5%~1%	20151	0.9998	0.9946	0.9978	0.9938	0.9958
	1%~10%	54011	0.9983	0.9907	0.9964	0.9910	0.9937
	10%~20%	19838	0.9978	0.9953	0.9971	0.9966	0.9969
	20%~30%	15717	0.9977	0.9957	0.9975	0.9975	0.9975
	30%~40%	13401	0.9977	0.9954	0.9976	0.9976	0.9976
	40%~50%	12757	0.9977	0.9953	0.9977	0.9977	0.9977
	Overall	188399	0.9987	0.9962	0.9976	0.9967	0.9972
15% Missing	MAF	#SNPs	Accuracy	r^2	Precision	Recall	F_1
	0.1%~0.5%	52524	0.9995	0.9674	0.9962	0.9580	0.9764
	0.5%~1%	20151	0.9982	0.9535	0.9936	0.9354	0.9627
	1%~10%	54011	0.9950	0.9721	0.9896	0.9726	0.9809
	10%~20%	19838	0.9931	0.9855	0.9913	0.9895	0.9904
	20%~30%	15717	0.9929	0.9868	0.9924	0.9922	0.9923
	30%~40%	13401	0.9929	0.9861	0.9928	0.9928	0.9928
	40%~50%	12757	0.9929	0.9855	0.9929	0.9929	0.9929
	Overall	188399	0.9959	0.9876	0.9927	0.9892	0.9910
25% Missing	MAF	#SNPs	Accuracy	r^2	Precision	Recall	F_1
	0.1%~0.5%	52524	0.9991	0.9392	0.9940	0.9193	0.9536
	0.5%~1%	20151	0.9972	0.9267	0.9897	0.8979	0.9390
	1%~10%	54011	0.9916	0.9529	0.9827	0.9531	0.9674
	10%~20%	19838	0.9879	0.9745	0.9850	0.9813	0.9832
	20%~30%	15717	0.9874	0.9766	0.9865	0.9860	0.9863
	30%~40%	13401	0.9873	0.9754	0.9872	0.9872	0.9872
	40%~50%	12757	0.9875	0.9746	0.9875	0.9875	0.9875
	Overall	188399	0.9930	0.9786	0.9875	0.9811	0.9843
50% Missing	MAF	#SNPs	Accuracy	r^2	Precision	Recall	F_1
	0.1%~0.5%	52524	0.9976	0.8366	0.9872	0.7786	0.8548
	0.5%~1%	20151	0.9937	0.8384	0.9782	0.7700	0.8451
	1%~10%	54011	0.9802	0.8893	0.9629	0.8843	0.9196
	10%~20%	19838	0.9675	0.9315	0.9630	0.9467	0.9546
	20%~30%	15717	0.9650	0.9344	0.9644	0.9596	0.9619
	30%~40%	13401	0.9647	0.9313	0.9649	0.9638	0.9644
	40%~50%	12757	0.9650	0.9288	0.9650	0.9649	0.9650
	Overall	188399	0.9818	0.9441	0.9694	0.9487	0.9587

Table S19. STICI's performance on the Caucasian cohort of LOS dataset

	MAF	#SNPs	Accuracy	r^2	Precision	Recall	F_1
5% Missing	**0.1%~0.5%**	**52524**	0.9968	0.7786	0.8809	0.7701	0.8129
	**0.5%~1%**	**20151**	0.9945	0.8566	0.9215	0.8313	0.8697
	**1%~10%**	**54011**	0.9891	0.9380	0.9649	0.9406	0.9522
	**10%~20%**	**19838**	0.9835	0.9642	0.9764	0.9737	0.9750
	**20%~30%**	**15717**	0.9793	0.9610	0.9776	0.9757	0.9766
	**30%~40%**	**13401**	0.9783	0.9591	0.9780	0.9775	0.9777
	**40%~50%**	**12757**	0.9774	0.9545	0.9775	0.9773	0.9774
	**Overall**	**188399**	0.9889	0.9657	0.9767	0.9695	0.9730
15% Missing	**MAF**	**#SNPs**	**Accuracy**	**r^2**	**Precision**	**Recall**	**F_1**
	**0.1%~0.5%**	**52524**	0.9974	0.8188	0.9303	0.7869	0.8427
	**0.5%~1%**	**20151**	0.9950	0.8691	0.9482	0.8325	0.8810
	**1%~10%**	**54011**	0.9899	0.9422	0.9730	0.9443	0.9581
	**10%~20%**	**19838**	0.9852	0.9674	0.9807	0.9768	0.9787
	**20%~30%**	**15717**	0.9827	0.9663	0.9818	0.9804	0.9811
	**30%~40%**	**13401**	0.9821	0.9643	0.9820	0.9817	0.9819
	**40%~50%**	**12757**	0.9820	0.9615	0.9820	0.9820	0.9820
	**Overall**	**188399**	0.9903	0.9696	0.9819	0.9738	0.9778
25% Missing	**MAF**	**#SNPs**	**Accuracy**	**r^2**	**Precision**	**Recall**	**F_1**
	**0.1%~0.5%**	**52524**	0.9975	0.8321	0.9515	0.7876	0.8506
	**0.5%~1%**	**20151**	0.9950	0.8721	0.9600	0.8291	0.8832
	**1%~10%**	**54011**	0.9890	0.9374	0.9747	0.9384	0.9557
	**10%~20%**	**19838**	0.9840	0.9647	0.9804	0.9750	0.9776
	**20%~30%**	**15717**	0.9822	0.9651	0.9815	0.9800	0.9807
	**30%~40%**	**13401**	0.9817	0.9631	0.9817	0.9815	0.9816
	**40%~50%**	**12757**	0.9818	0.9610	0.9818	0.9818	0.9818
	**Overall**	**188399**	0.9899	0.9683	0.9823	0.9727	0.9774
50% Missing	**MAF**	**#SNPs**	**Accuracy**	**r^2**	**Precision**	**Recall**	**F_1**
	**0.1%~0.5%**	**52524**	0.9970	0.8000	0.9717	0.7285	0.8082
	**0.5%~1%**	**20151**	0.9936	0.8343	0.9695	0.7656	0.8393
	**1%~10%**	**54011**	0.9844	0.9114	0.9710	0.9095	0.9380
	**10%~20%**	**19838**	0.9775	0.9510	0.9736	0.9650	0.9692
	**20%~30%**	**15717**	0.9764	0.9545	0.9752	0.9740	0.9746
	**30%~40%**	**13401**	0.9758	0.9518	0.9756	0.9758	0.9757
	**40%~50%**	**12757**	0.9763	0.9507	0.9762	0.9764	0.9763
	**Overall**	**188399**	0.9863	0.9575	0.9771	0.9628	0.9698

Table S20. GenoBERT's performance on the Caucasian cohort of LOS dataset

	MAF	#SNPs	Accuracy	r^2	Precision	Recall	F_1
5% Missing	0.1%~0.5%	52524	0.9997	0.9627	0.9993	0.9718	0.9853
	0.5%~1%	20151	0.9991	0.9584	0.9987	0.9692	0.9836
	1%~10%	54011	0.9977	0.9810	0.9980	0.9854	0.9917
	10%~20%	19838	0.9978	0.9929	0.9982	0.9961	0.9971
	20%~30%	15717	0.9977	0.9933	0.9981	0.9972	0.9976
	30%~40%	13401	0.9977	0.9935	0.9979	0.9976	0.9978
	40%~50%	12757	0.9975	0.9926	0.9976	0.9975	0.9976
	Overall	188399	0.9984	0.9926	0.9986	0.9951	0.9968
15% Missing	MAF	#SNPs	Accuracy	r^2	Precision	Recall	F_1
	0.1%~0.5%	52524	0.9995	0.9541	0.9982	0.9531	0.9748
	0.5%~1%	20151	0.9984	0.9469	0.9967	0.9400	0.9669
	1%~10%	54011	0.9966	0.9772	0.9952	0.9800	0.9875
	10%~20%	19838	0.9961	0.9902	0.9957	0.9940	0.9949
	20%~30%	15717	0.9960	0.9909	0.9960	0.9956	0.9958
	30%~40%	13401	0.9959	0.9906	0.9960	0.9959	0.9959
	40%~50%	12757	0.9958	0.9897	0.9959	0.9958	0.9958
	Overall	188399	0.9974	0.9904	0.9964	0.9927	0.9946
25% Missing	MAF	#SNPs	Accuracy	r^2	Precision	Recall	F_1
	0.1%~0.5%	52524	0.9992	0.9386	0.9964	0.9272	0.9594
	0.5%~1%	20151	0.9977	0.9342	0.9945	0.9166	0.9525
	1%~10%	54011	0.9949	0.9688	0.9920	0.9708	0.9812
	10%~20%	19838	0.9939	0.9856	0.9929	0.9908	0.9919
	20%~30%	15717	0.9937	0.9867	0.9935	0.9932	0.9933
	30%~40%	13401	0.9934	0.9857	0.9934	0.9935	0.9935
	40%~50%	12757	0.9934	0.9848	0.9934	0.9934	0.9934
	Overall	188399	0.9960	0.9864	0.9939	0.9892	0.9915
50% Missing	MAF	#SNPs	Accuracy	r^2	Precision	Recall	F_1
	0.1%~0.5%	52524	0.9977	0.8191	0.9731	0.7971	0.8664
	0.5%~1%	20151	0.9946	0.8442	0.9788	0.8086	0.8768
	1%~10%	54011	0.9878	0.9246	0.9808	0.9288	0.9534
	10%~20%	19838	0.9836	0.9606	0.9821	0.9742	0.9781
	20%~30%	15717	0.9824	0.9621	0.9826	0.9804	0.9815
	30%~40%	13401	0.9816	0.9590	0.9820	0.9815	0.9817
	40%~50%	12757	0.9815	0.9564	0.9816	0.9815	0.9816
	Overall	188399	0.9895	0.9638	0.9845	0.9710	0.9777

Models' performance on 1KGP/mixed populations cohort

Table S21. Beagle's performance on the mixed populations of 1KGP dataset

	MAF	#SNPs	Accuracy	r^2	Precision	Recall	F_1
**5% Missing**	**0.1%~0.5%**	**62625**	0.9961	0.8989	0.7477	0.7485	0.7480
	**0.5%~1%**	**41501**	0.9927	0.8981	0.7489	0.7494	0.7491
	**1%~10%**	**90533**	0.9703	0.9122	0.7499	0.7497	0.7498
	**10%~20%**	**17410**	0.8831	0.9095	0.7501	0.7500	0.7500
	**20%~30%**	**11479**	0.8238	0.8758	0.7484	0.7484	0.7484
	**30%~40%**	**9555**	0.7859	0.8506	0.7482	0.7482	0.7482
	**40%~50%**	**8010**	0.7666	0.8305	0.7484	0.7485	0.7485
	**Overall**	**241113**	0.9535	0.9212	0.7491	0.7491	0.7491
**15% Missing**	**MAF**	**#SNPs**	**Accuracy**	**r^2**	**Precision**	**Recall**	**F_1**
	**0.1%~0.5%**	**62625**	0.9960	0.8925	0.7454	0.7457	0.7455
	**0.5%~1%**	**41501**	0.9924	0.8890	0.7444	0.7430	0.7437
	**1%~10%**	**90533**	0.9699	0.9085	0.7484	0.7474	0.7479
	**10%~20%**	**17410**	0.8828	0.9079	0.7500	0.7498	0.7499
	**20%~30%**	**11479**	0.8244	0.8746	0.7493	0.7495	0.7494
	**30%~40%**	**9555**	0.7860	0.8491	0.7483	0.7485	0.7484
	**40%~50%**	**8010**	0.7671	0.8292	0.7488	0.7491	0.7489
	**Overall**	**241113**	0.9533	0.9194	0.7490	0.7487	0.7488
**25% Missing**	**MAF**	**#SNPs**	**Accuracy**	**r^2**	**Precision**	**Recall**	**F_1**
	**0.1%~0.5%**	**62625**	0.9958	0.8698	0.7411	0.7318	0.7363
	**0.5%~1%**	**41501**	0.9922	0.8778	0.7414	0.7367	0.7390
	**1%~10%**	**90533**	0.9691	0.9033	0.7447	0.7425	0.7436
	**10%~20%**	**17410**	0.8815	0.9052	0.7479	0.7474	0.7477
	**20%~30%**	**11479**	0.8221	0.8712	0.7462	0.7464	0.7463
	**30%~40%**	**9555**	0.7849	0.8463	0.7468	0.7473	0.7470
	**40%~50%**	**8010**	0.7663	0.8264	0.7476	0.7483	0.7480
	**Overall**	**241113**	0.9527	0.9163	0.7468	0.7458	0.7463
**50% Missing**	**MAF**	**#SNPs**	**Accuracy**	**r^2**	**Precision**	**Recall**	**F_1**
	**0.1%~0.5%**	**62625**	0.9951	0.8066	0.7336	0.6951	0.7124
	**0.5%~1%**	**41501**	0.9915	0.8428	0.7354	0.7172	0.7258
	**1%~10%**	**90533**	0.9674	0.8861	0.7387	0.7304	0.7344
	**10%~20%**	**17410**	0.8769	0.8936	0.7412	0.7390	0.7400
	**20%~30%**	**11479**	0.8166	0.8581	0.7388	0.7394	0.7390
	**30%~40%**	**9555**	0.7779	0.8322	0.7374	0.7396	0.7384
	**40%~50%**	**8010**	0.7593	0.8115	0.7381	0.7411	0.7396
	**Overall**	**241113**	0.9506	0.9048	0.7407	0.7365	0.7385

Table S22. SCDA's performance on the mixed populations of 1KGP dataset

	MAF	#SNPs	Accuracy	r^2	Precision	Recall	F_1
5% Missing	**0.1%~0.5%**	**62625**	0.9945	0.6350	0.8414	0.7521	0.7891
	**0.5%~1%**	**41501**	0.9914	0.6813	0.8868	0.7726	0.8200
	**1%~10%**	**90533**	0.9839	0.8691	0.9401	0.9045	0.9217
	**10%~20%**	**17410**	0.9748	0.9465	0.9591	0.9709	0.9649
	**20%~30%**	**11479**	0.9710	0.9428	0.9642	0.9736	0.9687
	**30%~40%**	**9555**	0.9704	0.9411	0.9681	0.9730	0.9703
	**40%~50%**	**8010**	0.9691	0.9352	0.9684	0.9702	0.9690
	**Overall**	**241113**	0.9857	0.9332	0.9567	0.9523	0.9545
15% Missing	**MAF**	**#SNPs**	**Accuracy**	**r^2**	**Precision**	**Recall**	**F_1**
	**0.1%~0.5%**	**62625**	0.9944	0.6246	0.8287	0.7590	0.7886
	**0.5%~1%**	**41501**	0.9907	0.6525	0.8723	0.7557	0.8034
	**1%~10%**	**90533**	0.9813	0.8458	0.9368	0.8815	0.9075
	**10%~20%**	**17410**	0.9692	0.9322	0.9566	0.9568	0.9567
	**20%~30%**	**11479**	0.9650	0.9285	0.9603	0.9635	0.9619
	**30%~40%**	**9555**	0.9644	0.9270	0.9630	0.9654	0.9641
	**40%~50%**	**8010**	0.9629	0.9196	0.9623	0.9635	0.9628
	**Overall**	**241113**	0.9834	0.9207	0.9545	0.9388	0.9465
25% Missing	**MAF**	**#SNPs**	**Accuracy**	**r^2**	**Precision**	**Recall**	**F_1**
	**0.1%~0.5%**	**62625**	0.9934	0.5542	0.7875	0.7119	0.7421
	**0.5%~1%**	**41501**	0.9896	0.6051	0.8470	0.7262	0.7739
	**1%~10%**	**90533**	0.9773	0.8097	0.9302	0.8479	0.8852
	**10%~20%**	**17410**	0.9586	0.9043	0.9511	0.9327	0.9417
	**20%~30%**	**11479**	0.9529	0.8990	0.9524	0.9449	0.9486
	**30%~40%**	**9555**	0.9520	0.8958	0.9529	0.9505	0.9517
	**40%~50%**	**8010**	0.9499	0.8856	0.9503	0.9497	0.9500
	**Overall**	**241113**	0.9792	0.8967	0.9499	0.9154	0.9320
50% Missing	**MAF**	**#SNPs**	**Accuracy**	**r^2**	**Precision**	**Recall**	**F_1**
	**0.1%~0.5%**	**62625**	0.9884	0.2546	0.5496	0.5863	0.5608
	**0.5%~1%**	**41501**	0.9835	0.3532	0.6662	0.6244	0.6369
	**1%~10%**	**90533**	0.9578	0.5985	0.8621	0.7027	0.7657
	**10%~20%**	**17410**	0.8819	0.6853	0.9080	0.7761	0.8293
	**20%~30%**	**11479**	0.8487	0.6417	0.8965	0.7969	0.8350
	**30%~40%**	**9555**	0.8298	0.6093	0.8800	0.8111	0.8324
	**40%~50%**	**8010**	0.8246	0.5888	0.8704	0.8188	0.8301
	**Overall**	**241113**	0.9500	0.6970	0.9080	0.7705	0.8292

Table S23. BiU-Net's performance on the mixed populations of 1KGP dataset

	MAF	#SNPs	Accuracy	r^2	Precision	Recall	F_1
5% Missing	**0.1%~0.5%**	**62625**	1.0000	0.9966	0.9984	0.9984	0.9984
	**0.5%~1%**	**41501**	0.9999	0.9965	0.9975	0.9989	0.9982
	**1%~10%**	**90533**	0.9989	0.9915	0.9964	0.9928	0.9946
	**10%~20%**	**17410**	0.9977	0.9952	0.9967	0.9965	0.9966
	**20%~30%**	**11479**	0.9973	0.9947	0.9969	0.9970	0.9969
	**30%~40%**	**9555**	0.9974	0.9948	0.9973	0.9973	0.9973
	**40%~50%**	**8010**	0.9972	0.9943	0.9972	0.9972	0.9972
	**Overall**	**241113**	0.9991	0.9957	0.9972	0.9964	0.9968
15% Missing	**MAF**	**#SNPs**	**Accuracy**	**r^2**	**Precision**	**Recall**	**F_1**
	**0.1%~0.5%**	**62625**	0.9995	0.9655	0.9952	0.9703	0.9824
	**0.5%~1%**	**41501**	0.9982	0.9344	0.9917	0.9437	0.9665
	**1%~10%**	**90533**	0.9961	0.9698	0.9891	0.9730	0.9810
	**10%~20%**	**17410**	0.9927	0.9849	0.9899	0.9889	0.9894
	**20%~30%**	**11479**	0.9915	0.9836	0.9904	0.9904	0.9904
	**30%~40%**	**9555**	0.9915	0.9836	0.9912	0.9913	0.9913
	**40%~50%**	**8010**	0.9912	0.9821	0.9911	0.9912	0.9911
	**Overall**	**241113**	0.9965	0.9844	0.9911	0.9856	0.9883
25% Missing	**MAF**	**#SNPs**	**Accuracy**	**r^2**	**Precision**	**Recall**	**F_1**
	**0.1%~0.5%**	**62625**	0.9977	0.8495	0.9903	0.8582	0.9142
	**0.5%~1%**	**41501**	0.9962	0.8619	0.9845	0.8778	0.9248
	**1%~10%**	**90533**	0.9928	0.9443	0.9809	0.9492	0.9645
	**10%~20%**	**17410**	0.9866	0.9720	0.9819	0.9792	0.9806
	**20%~30%**	**11479**	0.9844	0.9699	0.9826	0.9822	0.9824
	**30%~40%**	**9555**	0.9844	0.9697	0.9839	0.9841	0.9840
	**40%~50%**	**8010**	0.9838	0.9669	0.9837	0.9838	0.9837
	**Overall**	**241113**	0.9932	0.9694	0.9840	0.9706	0.9772
50% Missing	**MAF**	**#SNPs**	**Accuracy**	**r^2**	**Precision**	**Recall**	**F_1**
	**0.1%~0.5%**	**62625**	0.9949	0.6621	0.9731	0.6770	0.7594
	**0.5%~1%**	**41501**	0.9921	0.7132	0.9653	0.7406	0.8188
	**1%~10%**	**90533**	0.9827	0.8651	0.9576	0.8725	0.9108
	**10%~20%**	**17410**	0.9639	0.9236	0.9550	0.9403	0.9475
	**20%~30%**	**11479**	0.9568	0.9147	0.9539	0.9490	0.9514
	**30%~40%**	**9555**	0.9561	0.9127	0.9556	0.9546	0.9551
	**40%~50%**	**8010**	0.9546	0.9053	0.9545	0.9543	0.9544
	**Overall**	**241113**	0.9829	0.9218	0.9606	0.9238	0.9414

Table S24. STICI's performance on the mixed populations of 1KGP dataset

	MAF	#SNPs	Accuracy	r^2	Precision	Recall	F_1
**5% Missing**	**0.1%~0.5%**	**62625**	0.9943	0.5955	0.8671	0.6642	0.7239
	**0.5%~1%**	**41501**	0.9922	0.6983	0.9037	0.7512	0.8072
	**1%~10%**	**90533**	0.9843	0.8594	0.9481	0.8785	0.9100
	**10%~20%**	**17410**	0.9678	0.9101	0.9603	0.9416	0.9506
	**20%~30%**	**11479**	0.9610	0.8988	0.9586	0.9499	0.9541
	**30%~40%**	**9555**	0.9560	0.8846	0.9574	0.9521	0.9545
	**40%~50%**	**8010**	0.9528	0.8657	0.9542	0.9519	0.9528
	**Overall**	**241113**	0.9838	0.9089	0.9596	0.9227	0.9402
**15% Missing**	**MAF**	**#SNPs**	**Accuracy**	**r^2**	**Precision**	**Recall**	**F_1**
	**0.1%~0.5%**	**62625**	0.9954	0.6740	0.9120	0.7200	0.7846
	**0.5%~1%**	**41501**	0.9933	0.7427	0.9286	0.7850	0.8409
	**1%~10%**	**90533**	0.9869	0.8840	0.9597	0.8998	0.9276
	**10%~20%**	**17410**	0.9732	0.9283	0.9671	0.9530	0.9599
	**20%~30%**	**11479**	0.9674	0.9198	0.9657	0.9593	0.9624
	**30%~40%**	**9555**	0.9648	0.9119	0.9655	0.9623	0.9639
	**40%~50%**	**8010**	0.9623	0.8991	0.9630	0.9618	0.9623
	**Overall**	**241113**	0.9866	0.9273	0.9676	0.9374	0.9519
**25% Missing**	**MAF**	**#SNPs**	**Accuracy**	**r^2**	**Precision**	**Recall**	**F_1**
	**0.1%~0.5%**	**62625**	0.9952	0.6693	0.9309	0.7042	0.7766
	**0.5%~1%**	**41501**	0.9932	0.7401	0.9406	0.7780	0.8406
	**1%~10%**	**90533**	0.9865	0.8837	0.9633	0.8971	0.9277
	**10%~20%**	**17410**	0.9719	0.9287	0.9668	0.9512	0.9588
	**20%~30%**	**11479**	0.9661	0.9202	0.9650	0.9581	0.9615
	**30%~40%**	**9555**	0.9641	0.9142	0.9649	0.9619	0.9633
	**40%~50%**	**8010**	0.9620	0.9044	0.9626	0.9615	0.9620
	**Overall**	**241113**	0.9862	0.9279	0.9687	0.9359	0.9517
**50% Missing**	**MAF**	**#SNPs**	**Accuracy**	**r^2**	**Precision**	**Recall**	**F_1**
	**0.1%~0.5%**	**62625**	0.9944	0.6203	0.9553	0.6398	0.7165
	**0.5%~1%**	**41501**	0.9917	0.6911	0.9551	0.7225	0.8007
	**1%~10%**	**90533**	0.9820	0.8515	0.9604	0.8608	0.9048
	**10%~20%**	**17410**	0.9615	0.9111	0.9562	0.9328	0.9440
	**20%~30%**	**11479**	0.9533	0.8996	0.9521	0.9430	0.9474
	**30%~40%**	**9555**	0.9519	0.8960	0.9523	0.9496	0.9509
	**40%~50%**	**8010**	0.9496	0.8866	0.9498	0.9491	0.9494
	**Overall**	**241113**	0.9818	0.9108	0.9607	0.9152	0.9368

Table S25. GenoBERT's performance on the mixed populations of 1KGP dataset

	MAF	#SNPs	Accuracy	r^2	Precision	Recall	F_1
5% Missing	**0.1%~0.5%**	**62625**	1.0000	0.9947	0.9992	0.9975	0.9984
	**0.5%~1%**	**41501**	0.9999	0.9946	0.9990	0.9975	0.9983
	**1%~10%**	**90533**	0.9993	0.9935	0.9988	0.9945	0.9967
	**10%~20%**	**17410**	0.9985	0.9961	0.9986	0.9973	0.9980
	**20%~30%**	**11479**	0.9983	0.9958	0.9985	0.9978	0.9981
	**30%~40%**	**9555**	0.9983	0.9961	0.9985	0.9982	0.9983
	**40%~50%**	**8010**	0.9983	0.9958	0.9983	0.9983	0.9983
	**Overall**	**241113**	0.9994	0.9966	0.9989	0.9972	0.9981
15% Missing	**MAF**	**#SNPs**	**Accuracy**	**r^2**	**Precision**	**Recall**	**F_1**
	**0.1%~0.5%**	**62625**	0.9996	0.9622	0.9968	0.9715	0.9840
	**0.5%~1%**	**41501**	0.9987	0.9456	0.9960	0.9564	0.9756
	**1%~10%**	**90533**	0.9976	0.9779	0.9961	0.9811	0.9885
	**10%~20%**	**17410**	0.9955	0.9884	0.9956	0.9921	0.9939
	**20%~30%**	**11479**	0.9948	0.9875	0.9953	0.9935	0.9944
	**30%~40%**	**9555**	0.9949	0.9882	0.9952	0.9946	0.9949
	**40%~50%**	**8010**	0.9948	0.9873	0.9949	0.9947	0.9948
	**Overall**	**241113**	0.9978	0.9881	0.9964	0.9897	0.9930
25% Missing	**MAF**	**#SNPs**	**Accuracy**	**r^2**	**Precision**	**Recall**	**F_1**
	**0.1%~0.5%**	**62625**	0.9980	0.8508	0.9913	0.8705	0.9237
	**0.5%~1%**	**41501**	0.9973	0.8860	0.9911	0.9067	0.9457
	**1%~10%**	**90533**	0.9955	0.9593	0.9922	0.9654	0.9785
	**10%~20%**	**17410**	0.9917	0.9789	0.9916	0.9859	0.9887
	**20%~30%**	**11479**	0.9904	0.9770	0.9911	0.9882	0.9897
	**30%~40%**	**9555**	0.9906	0.9781	0.9911	0.9901	0.9906
	**40%~50%**	**8010**	0.9903	0.9763	0.9906	0.9902	0.9904
	**Overall**	**241113**	0.9956	0.9765	0.9929	0.9793	0.9860
50% Missing	**MAF**	**#SNPs**	**Accuracy**	**r^2**	**Precision**	**Recall**	**F_1**
	**0.1%~0.5%**	**62625**	0.9947	0.5882	0.9172	0.6825	0.7599
	**0.5%~1%**	**41501**	0.9931	0.7018	0.9489	0.7786	0.8479
	**1%~10%**	**90533**	0.9870	0.8771	0.9730	0.9033	0.9359
	**10%~20%**	**17410**	0.9724	0.9244	0.9739	0.9523	0.9628
	**20%~30%**	**11479**	0.9673	0.9170	0.9713	0.9593	0.9651
	**30%~40%**	**9555**	0.9663	0.9148	0.9693	0.9642	0.9666
	**40%~50%**	**8010**	0.9653	0.9082	0.9669	0.9647	0.9656
	**Overall**	**241113**	0.9865	0.9233	0.9768	0.9384	0.9569

Models' performance on 1KGP/EUR cohort

Table S26. STICI's performance on the European cohort of 1KGP dataset

	MAF	#SNPs	Accuracy	r^2	Precision	Recall	F_1
5% Missing	**0.1%~0.5%**	**3664**	0.9819	0.5288	0.8590	0.6348	0.6905
	**0.5%~1%**	**4519**	0.9810	0.7666	0.8789	0.7080	0.7649
	**1%~10%**	**45930**	0.9798	0.9050	0.9439	0.9028	0.9219
	**10%~20%**	**21922**	0.9763	0.9500	0.9663	0.9579	0.9620
	**20%~30%**	**16560**	0.9739	0.9528	0.9711	0.9684	0.9697
	**30%~40%**	**14516**	0.9718	0.9470	0.9712	0.9703	0.9707
	**40%~50%**	**13466**	0.9709	0.9442	0.9708	0.9707	0.9708
	**Overall**	**120577**	0.9765	0.9501	0.9672	0.9573	0.9621
15% Missing	**MAF**	**#SNPs**	**Accuracy**	**r^2**	**Precision**	**Recall**	**F_1**
	**0.1%~0.5%**	**3664**	0.9823	0.5354	0.8629	0.6435	0.7005
	**0.5%~1%**	**4519**	0.9819	0.7772	0.8872	0.7224	0.7792
	**1%~10%**	**45930**	0.9792	0.9021	0.9456	0.9007	0.9216
	**10%~20%**	**21922**	0.9761	0.9487	0.9671	0.9583	0.9626
	**20%~30%**	**16560**	0.9743	0.9531	0.9719	0.9694	0.9706
	**30%~40%**	**14516**	0.9729	0.9492	0.9724	0.9717	0.9720
	**40%~50%**	**13466**	0.9724	0.9468	0.9723	0.9723	0.9723
	**Overall**	**120577**	0.9766	0.9501	0.9684	0.9582	0.9632
25% Missing	**MAF**	**#SNPs**	**Accuracy**	**r^2**	**Precision**	**Recall**	**F_1**
	**0.1%~0.5%**	**3664**	0.9819	0.5205	0.8752	0.6314	0.6899
	**0.5%~1%**	**4519**	0.9805	0.7596	0.8913	0.6938	0.7565
	**1%~10%**	**45930**	0.9771	0.8912	0.9463	0.8898	0.9158
	**10%~20%**	**21922**	0.9743	0.9439	0.9663	0.9560	0.9611
	**20%~30%**	**16560**	0.9732	0.9507	0.9710	0.9687	0.9698
	**30%~40%**	**14516**	0.9725	0.9479	0.9720	0.9715	0.9718
	**40%~50%**	**13466**	0.9720	0.9449	0.9719	0.9719	0.9719
	**Overall**	**120577**	0.9752	0.9464	0.9679	0.9562	0.9619
50% Missing	**MAF**	**#SNPs**	**Accuracy**	**r^2**	**Precision**	**Recall**	**F_1**
	**0.1%~0.5%**	**3664**	0.9839	0.5700	0.9238	0.6638	0.7334
	**0.5%~1%**	**4519**	0.9826	0.7847	0.9269	0.7187	0.7870
	**1%~10%**	**45930**	0.9738	0.8739	0.9475	0.8720	0.9059
	**10%~20%**	**21922**	0.9665	0.9251	0.9594	0.9437	0.9513
	**20%~30%**	**16560**	0.9659	0.9367	0.9637	0.9613	0.9625
	**30%~40%**	**14516**	0.9665	0.9354	0.9660	0.9659	0.9659
	**40%~50%**	**13466**	0.9666	0.9324	0.9666	0.9666	0.9666
	**Overall**	**120577**	0.9703	0.9348	0.9631	0.9487	0.9557

Table S27. GenoBERT's performance on the European cohort of 1KGP dataset

	MAF	#SNPs	Accuracy	r^2	Precision	Recall	F_1
5% Missing	0.1%~0.5%	3664	0.9992	0.9780	0.9950	0.9854	0.9901
	0.5%~1%	4519	0.9993	0.9865	0.9948	0.9929	0.9939
	1%~10%	45930	0.9989	0.9938	0.9964	0.9964	0.9964
	10%~20%	21922	0.9975	0.9938	0.9973	0.9957	0.9965
	20%~30%	16560	0.9975	0.9946	0.9976	0.9970	0.9973
	30%~40%	14516	0.9971	0.9936	0.9973	0.9970	0.9971
	40%~50%	13466	0.9970	0.9929	0.9970	0.9970	0.9970
	Overall	120577	0.9980	0.9951	0.9976	0.9967	0.9972
15% Missing	MAF	#SNPs	Accuracy	r^2	Precision	Recall	F_1
	0.1%~0.5%	3664	0.9990	0.9729	0.9930	0.9820	0.9874
	0.5%~1%	4519	0.9987	0.9765	0.9925	0.9840	0.9882
	1%~10%	45930	0.9957	0.9775	0.9915	0.9813	0.9863
	10%~20%	21922	0.9948	0.9875	0.9941	0.9916	0.9928
	20%~30%	16560	0.9948	0.9890	0.9948	0.9941	0.9945
	30%~40%	14516	0.9948	0.9885	0.9949	0.9946	0.9948
	40%~50%	13466	0.9945	0.9872	0.9946	0.9945	0.9945
	Overall	120577	0.9954	0.9887	0.9947	0.9923	0.9935
25% Missing	MAF	#SNPs	Accuracy	r^2	Precision	Recall	F_1
	0.1%~0.5%	3664	0.9953	0.8704	0.9898	0.9020	0.9417
	0.5%~1%	4519	0.9930	0.9034	0.9881	0.8867	0.9318
	1%~10%	45930	0.9913	0.9555	0.9856	0.9591	0.9720
	10%~20%	21922	0.9916	0.9799	0.9902	0.9866	0.9884
	20%~30%	16560	0.9919	0.9835	0.9916	0.9910	0.9913
	30%~40%	14516	0.9922	0.9832	0.9923	0.9921	0.9922
	40%~50%	13466	0.9919	0.9818	0.9919	0.9919	0.9919
	Overall	120577	0.9918	0.9806	0.9910	0.9861	0.9885
50% Missing	MAF	#SNPs	Accuracy	r^2	Precision	Recall	F_1
	0.1%~0.5%	3664	0.9942	0.8381	0.9723	0.8894	0.9261
	0.5%~1%	4519	0.9908	0.8695	0.9719	0.8585	0.9077
	1%~10%	45930	0.9837	0.9174	0.9700	0.9246	0.9461
	10%~20%	21922	0.9812	0.9559	0.9780	0.9699	0.9739
	20%~30%	16560	0.9816	0.9636	0.9810	0.9793	0.9802
	30%~40%	14516	0.9819	0.9622	0.9820	0.9816	0.9818
	40%~50%	13466	0.9817	0.9598	0.9817	0.9817	0.9817
	Overall	120577	0.9831	0.9605	0.9803	0.9716	0.9759

Models' performance on 1KGP/AFR cohort

Table S28. STICI's performance on the African cohort of 1KGP dataset

	MAF	#SNPs	Accuracy	r^2	Precision	Recall	F_1
5% Missing	0.1%~0.5%	7140	0.9839	0.5652	0.8876	0.6572	0.7209
	0.5%~1%	10402	0.9850	0.8197	0.9142	0.7520	0.8118
	1%~10%	100338	0.9797	0.8896	0.9344	0.8869	0.9087
	10%~20%	34755	0.9640	0.9160	0.9441	0.9305	0.9369
	20%~30%	19414	0.9568	0.9174	0.9494	0.9450	0.9470
	30%~40%	14704	0.9522	0.9040	0.9506	0.9486	0.9494
	40%~50%	12851	0.9514	0.9007	0.9513	0.9509	0.9509
	Overall	199604	0.9713	0.9227	0.9475	0.9292	0.9378
15% Missing	MAF	#SNPs	Accuracy	r^2	Precision	Recall	F_1
	0.1%~0.5%	7140	0.9849	0.5906	0.9011	0.6788	0.7454
	0.5%~1%	10402	0.9861	0.8313	0.9222	0.7710	0.8289
	1%~10%	100338	0.9778	0.8770	0.9367	0.8779	0.9048
	10%~20%	34755	0.9618	0.9090	0.9447	0.9279	0.9359
	20%~30%	19414	0.9541	0.9107	0.9481	0.9427	0.9453
	30%~40%	14704	0.9506	0.9003	0.9493	0.9474	0.9482
	40%~50%	12851	0.9498	0.8970	0.9497	0.9493	0.9494
	Overall	199604	0.9696	0.9168	0.9479	0.9264	0.9367
25% Missing	MAF	#SNPs	Accuracy	r^2	Precision	Recall	F_1
	0.1%~0.5%	7140	0.9859	0.6161	0.9138	0.6982	0.7669
	0.5%~1%	10402	0.9868	0.8395	0.9291	0.7828	0.8401
	1%~10%	100338	0.9764	0.8679	0.9385	0.8716	0.9020
	10%~20%	34755	0.9580	0.8988	0.9432	0.9222	0.9323
	20%~30%	19414	0.9498	0.9017	0.9450	0.9381	0.9415
	30%~40%	14704	0.9466	0.8925	0.9456	0.9435	0.9445
	40%~50%	12851	0.9457	0.8885	0.9457	0.9453	0.9455
	Overall	199604	0.9673	0.9099	0.9468	0.9221	0.9339
50% Missing	MAF	#SNPs	Accuracy	r^2	Precision	Recall	F_1
	0.1%~0.5%	7140	0.9877	0.6612	0.9487	0.7348	0.8090
	0.5%~1%	10402	0.9862	0.8301	0.9483	0.7694	0.8359
	1%~10%	100338	0.9691	0.8255	0.9385	0.8319	0.8777
	10%~20%	34755	0.9398	0.8540	0.9290	0.8901	0.9083
	20%~30%	19414	0.9292	0.8602	0.9259	0.9147	0.9201
	30%~40%	14704	0.9266	0.8544	0.9259	0.9236	0.9247
	40%~50%	12851	0.9251	0.8466	0.9250	0.9248	0.9249
	Overall	199604	0.9557	0.8770	0.9346	0.8965	0.9145

Table S29. GenoBERT's performance on the African cohort of 1KGP dataset

	MAF	#SNPs	Accuracy	r^2	Precision	Recall	F_1
5% Missing	0.1%~0.5%	7140	0.9988	0.9589	0.9845	0.9832	0.9838
	0.5%~1%	10402	0.9989	0.9838	0.9860	0.9948	0.9903
	1%~10%	100338	0.9972	0.9832	0.9884	0.9926	0.9905
	10%~20%	34755	0.9935	0.9839	0.9915	0.9902	0.9908
	20%~30%	19414	0.9937	0.9874	0.9930	0.9933	0.9932
	30%~40%	14704	0.9935	0.9867	0.9935	0.9934	0.9935
	40%~50%	12851	0.9936	0.9861	0.9936	0.9936	0.9936
	Overall	199604	0.9959	0.9880	0.9922	0.9930	0.9926
15% Missing	MAF	#SNPs	Accuracy	r^2	Precision	Recall	F_1
	0.1%~0.5%	7140	0.9984	0.9475	0.9763	0.9808	0.9786
	0.5%~1%	10402	0.9978	0.9692	0.9756	0.9853	0.9804
	1%~10%	100338	0.9907	0.9467	0.9743	0.9634	0.9688
	10%~20%	34755	0.9852	0.9637	0.9802	0.9781	0.9792
	20%~30%	19414	0.9845	0.9689	0.9830	0.9832	0.9831
	30%~40%	14704	0.9844	0.9683	0.9841	0.9843	0.9842
	40%~50%	12851	0.9846	0.9675	0.9845	0.9846	0.9846
	Overall	199604	0.9889	0.9688	0.9817	0.9793	0.9805
25% Missing	MAF	#SNPs	Accuracy	r^2	Precision	Recall	F_1
	0.1%~0.5%	7140	0.9980	0.9369	0.9785	0.9693	0.9738
	0.5%~1%	10402	0.9964	0.9512	0.9718	0.9627	0.9672
	1%~10%	100338	0.9862	0.9212	0.9643	0.9424	0.9531
	10%~20%	34755	0.9777	0.9459	0.9705	0.9666	0.9686
	20%~30%	19414	0.9761	0.9529	0.9740	0.9739	0.9740
	30%~40%	14704	0.9759	0.9516	0.9756	0.9757	0.9756
	40%~50%	12851	0.9761	0.9500	0.9761	0.9761	0.9761
	Overall	199604	0.9833	0.9533	0.9731	0.9677	0.9704
50% Missing	MAF	#SNPs	Accuracy	r^2	Precision	Recall	F_1
	0.1%~0.5%	7140	0.9946	0.8375	0.9666	0.8989	0.9301
	0.5%~1%	10402	0.9905	0.8746	0.9619	0.8619	0.9057
	1%~10%	100338	0.9739	0.8502	0.9431	0.8770	0.9074
	10%~20%	34755	0.9561	0.8926	0.9461	0.9287	0.9372
	20%~30%	19414	0.9512	0.9031	0.9489	0.9442	0.9465
	30%~40%	14704	0.9495	0.8976	0.9493	0.9483	0.9488
	40%~50%	12851	0.9491	0.8936	0.9492	0.9490	0.9491
	Overall	199604	0.9668	0.9066	0.9510	0.9298	0.9401

Models' performance on 1KGP/AMR cohort

Table S30. STICI's performance on the admixed American cohort of 1KGP dataset

	MAF	#SNPs	Accuracy	r^2	Precision	Recall	F_1
5% Missing	0.1%~0.5%	9403	0.9811	0.6372	0.7590	0.6811	0.6991
	0.5%~1%	11925	0.9800	0.6837	0.7648	0.7314	0.7367
	1%~10%	82174	0.9710	0.8659	0.8591	0.8508	0.8535
	10%~20%	24826	0.9415	0.8979	0.8989	0.8965	0.8976
	20%~30%	16815	0.9270	0.8940	0.9093	0.9112	0.9102
	30%~40%	15134	0.9191	0.8699	0.9129	0.9139	0.9134
	40%~50%	12960	0.9191	0.8651	0.9172	0.9179	0.9175
	Overall	173237	0.9552	0.9059	0.9015	0.8988	0.9000
15% Missing	MAF	#SNPs	Accuracy	r^2	Precision	Recall	F_1
	0.1%~0.5%	9403	0.9823	0.6634	0.7831	0.6899	0.7136
	0.5%~1%	11925	0.9813	0.7031	0.7846	0.7370	0.7488
	1%~10%	82174	0.9687	0.8428	0.8626	0.8329	0.8457
	10%~20%	24826	0.9376	0.8838	0.8979	0.8889	0.8933
	20%~30%	16815	0.9232	0.8812	0.9074	0.9060	0.9067
	30%~40%	15134	0.9162	0.8607	0.9108	0.9107	0.9107
	40%~50%	12960	0.9162	0.8549	0.9144	0.9149	0.9146
	Overall	173237	0.9529	0.8951	0.9024	0.8917	0.8969
25% Missing	MAF	#SNPs	Accuracy	r^2	Precision	Recall	F_1
	0.1%~0.5%	9403	0.9833	0.6852	0.8090	0.6945	0.7268
	0.5%~1%	11925	0.9821	0.7046	0.8074	0.7329	0.7562
	1%~10%	82174	0.9669	0.8225	0.8686	0.8166	0.8395
	10%~20%	24826	0.9329	0.8692	0.8960	0.8795	0.8875
	20%~30%	16815	0.9192	0.8695	0.9052	0.9004	0.9028
	30%~40%	15134	0.9129	0.8524	0.9082	0.9070	0.9076
	40%~50%	12960	0.9125	0.8454	0.9109	0.9110	0.9110
	Overall	173237	0.9505	0.8850	0.9031	0.8843	0.8934
50% Missing	MAF	#SNPs	Accuracy	r^2	Precision	Recall	F_1
	0.1%~0.5%	9403	0.9838	0.6738	0.8597	0.6823	0.7344
	0.5%~1%	11925	0.9811	0.6382	0.8643	0.6856	0.7426
	1%~10%	82174	0.9598	0.7597	0.8850	0.7602	0.8109
	10%~20%	24826	0.9160	0.8238	0.8867	0.8458	0.8649
	20%~30%	16815	0.9012	0.8298	0.8904	0.8780	0.8839
	30%~40%	15134	0.8967	0.8193	0.8931	0.8897	0.8913
	40%~50%	12960	0.8954	0.8088	0.8941	0.8936	0.8938
	Overall	173237	0.9403	0.8503	0.8982	0.8559	0.8758

Table S31. GenoBERT's performance on the mixed American cohort of 1KGP dataset

	MAF	#SNPs	Accuracy	r^2	Precision	Recall	F_1
5% Missing	0.1%~0.5%	9403	0.9984	0.9509	0.9750	0.9769	0.9756
	0.5%~1%	11925	0.9985	0.9603	0.9778	0.9838	0.9805
	1%~10%	82174	0.9970	0.9772	0.9876	0.9890	0.9883
	10%~20%	24826	0.9908	0.9759	0.9911	0.9834	0.9872
	20%~30%	16815	0.9897	0.9771	0.9915	0.9868	0.9891
	30%~40%	15134	0.9879	0.9731	0.9897	0.9864	0.9880
	40%~50%	12960	0.9878	0.9713	0.9886	0.9873	0.9879
	Overall	173237	0.9941	0.9812	0.9922	0.9871	0.9896
15% Missing	MAF	#SNPs	Accuracy	r^2	Precision	Recall	F_1
	0.1%~0.5%	9403	0.9966	0.9114	0.9432	0.9779	0.9596
	0.5%~1%	11925	0.9961	0.9125	0.9453	0.9769	0.9603
	1%~10%	82174	0.9903	0.9366	0.9675	0.9620	0.9647
	10%~20%	24826	0.9825	0.9580	0.9803	0.9713	0.9758
	20%~30%	16815	0.9804	0.9599	0.9822	0.9760	0.9791
	30%~40%	15134	0.9784	0.9553	0.9806	0.9766	0.9785
	40%~50%	12960	0.9773	0.9497	0.9785	0.9766	0.9774
	Overall	173237	0.9870	0.9624	0.9811	0.9736	0.9773
25% Missing	MAF	#SNPs	Accuracy	r^2	Precision	Recall	F_1
	0.1%~0.5%	9403	0.9948	0.8619	0.9153	0.9662	0.9395
	0.5%~1%	11925	0.9928	0.8398	0.9144	0.9478	0.9304
	1%~10%	82174	0.9850	0.9031	0.9487	0.9424	0.9455
	10%~20%	24826	0.9771	0.9468	0.9704	0.9656	0.9680
	20%~30%	16815	0.9764	0.9533	0.9758	0.9733	0.9745
	30%~40%	15134	0.9760	0.9519	0.9765	0.9751	0.9758
	40%~50%	12960	0.9747	0.9455	0.9750	0.9744	0.9747
	Overall	173237	0.9826	0.9512	0.9712	0.9675	0.9693
50% Missing	MAF	#SNPs	Accuracy	r^2	Precision	Recall	F_1
	0.1%~0.5%	9403	0.9930	0.8051	0.9686	0.8704	0.9139
	0.5%~1%	11925	0.9884	0.7210	0.9546	0.8195	0.8761
	1%~10%	82174	0.9766	0.8439	0.9465	0.8753	0.9080
	10%~20%	24826	0.9628	0.9123	0.9561	0.9359	0.9457
	20%~30%	16815	0.9602	0.9202	0.9598	0.9517	0.9557
	30%~40%	15134	0.9600	0.9182	0.9608	0.9573	0.9590
	40%~50%	12960	0.9571	0.9066	0.9576	0.9562	0.9569
	Overall	173237	0.9718	0.9195	0.9606	0.9356	0.9477

Models' performance on 1KGP/SAS cohort

Table S32. STICI's performance on the South Asian cohort of 1KGP dataset

	MAF	#SNPs	Accuracy	r^2	Precision	Recall	F_1
5% Missing	0.1%~0.5%	3695	0.9797	0.7655	0.8847	0.6335	0.6952
	0.5%~1%	5229	0.9810	0.6896	0.8774	0.6990	0.7559
	1%~10%	49481	0.9820	0.9084	0.9504	0.9077	0.9275
	10%~20%	22056	0.9757	0.9446	0.9658	0.9565	0.9610
	20%~30%	17336	0.9732	0.9418	0.9702	0.9666	0.9684
	30%~40%	15352	0.9711	0.9335	0.9703	0.9690	0.9696
	40%~50%	13355	0.9704	0.9293	0.9703	0.9699	0.9700
	Overall	126504	0.9770	0.9433	0.9675	0.9561	0.9617
15% Missing	MAF	#SNPs	Accuracy	r^2	Precision	Recall	F_1
	0.1%~0.5%	3695	0.9804	0.7725	0.8926	0.6472	0.7119
	0.5%~1%	5229	0.9820	0.7076	0.8903	0.7162	0.7744
	1%~10%	49481	0.9810	0.9033	0.9503	0.9050	0.9262
	10%~20%	22056	0.9747	0.9425	0.9660	0.9561	0.9610
	20%~30%	17336	0.9730	0.9421	0.9703	0.9675	0.9689
	30%~40%	15352	0.9714	0.9358	0.9707	0.9698	0.9703
	40%~50%	13355	0.9712	0.9329	0.9711	0.9708	0.9710
	Overall	126504	0.9766	0.9430	0.9679	0.9567	0.9622
25% Missing	MAF	#SNPs	Accuracy	r^2	Precision	Recall	F_1
	0.1%~0.5%	3695	0.9807	0.7767	0.9017	0.6501	0.7168
	0.5%~1%	5229	0.9824	0.7170	0.8962	0.7215	0.7807
	1%~10%	49481	0.9803	0.9002	0.9505	0.9023	0.9247
	10%~20%	22056	0.9730	0.9393	0.9648	0.9540	0.9593
	20%~30%	17336	0.9718	0.9407	0.9691	0.9664	0.9678
	30%~40%	15352	0.9706	0.9353	0.9700	0.9693	0.9696
	40%~50%	13355	0.9706	0.9327	0.9705	0.9703	0.9704
	Overall	126504	0.9757	0.9416	0.9672	0.9558	0.9614
50% Missing	MAF	#SNPs	Accuracy	r^2	Precision	Recall	F_1
	0.1%~0.5%	3695	0.9817	0.7890	0.9275	0.6614	0.7327
	0.5%~1%	5229	0.9828	0.7271	0.9257	0.7192	0.7879
	1%~10%	49481	0.9751	0.8758	0.9486	0.8755	0.9085
	10%~20%	22056	0.9636	0.9190	0.9566	0.9396	0.9478
	20%~30%	17336	0.9634	0.9255	0.9606	0.9582	0.9594
	30%~40%	15352	0.9624	0.9211	0.9617	0.9616	0.9616
	40%~50%	13355	0.9633	0.9204	0.9631	0.9632	0.9632
	Overall	126504	0.9692	0.9278	0.9605	0.9456	0.9529

Table S33. GenoBERT's performance on the South Asian cohort of 1KGP dataset

	MAF	#SNPs	Accuracy	r^2	Precision	Recall	F_1
5% Missing	0.1%~0.5%	3695	0.9996	0.9941	0.9964	0.9967	0.9966
	0.5%~1%	5229	0.9996	0.9923	0.9953	0.9976	0.9965
	1%~10%	49481	0.9992	0.9950	0.9973	0.9974	0.9973
	10%~20%	22056	0.9976	0.9940	0.9978	0.9957	0.9968
	20%~30%	17336	0.9966	0.9925	0.9973	0.9956	0.9964
	30%~40%	15352	0.9951	0.9894	0.9955	0.9946	0.9951
	40%~50%	13355	0.9957	0.9897	0.9959	0.9955	0.9957
	Overall	126504	0.9977	0.9940	0.9976	0.9957	0.9967
15% Missing	MAF	#SNPs	Accuracy	r^2	Precision	Recall	F_1
	0.1%~0.5%	3695	0.9993	0.9881	0.9924	0.9935	0.9929
	0.5%~1%	5229	0.9991	0.9821	0.9903	0.9923	0.9913
	1%~10%	49481	0.9966	0.9815	0.9925	0.9858	0.9891
	10%~20%	22056	0.9947	0.9875	0.9945	0.9912	0.9928
	20%~30%	17336	0.9945	0.9878	0.9948	0.9934	0.9941
	30%~40%	15352	0.9937	0.9860	0.9941	0.9934	0.9938
	40%~50%	13355	0.9939	0.9855	0.9941	0.9938	0.9940
	Overall	126504	0.9955	0.9886	0.9950	0.9922	0.9936
25% Missing	MAF	#SNPs	Accuracy	r^2	Precision	Recall	F_1
	0.1%~0.5%	3695	0.9985	0.9785	0.9911	0.9800	0.9855
	0.5%~1%	5229	0.9980	0.9662	0.9879	0.9755	0.9816
	1%~10%	49481	0.9952	0.9745	0.9897	0.9791	0.9843
	10%~20%	22056	0.9932	0.9846	0.9925	0.9892	0.9908
	20%~30%	17336	0.9935	0.9862	0.9936	0.9925	0.9930
	30%~40%	15352	0.9932	0.9851	0.9934	0.9929	0.9932
	40%~50%	13355	0.9933	0.9846	0.9934	0.9932	0.9933
	Overall	126504	0.9944	0.9862	0.9934	0.9904	0.9919
50% Missing	MAF	#SNPs	Accuracy	r^2	Precision	Recall	F_1
	0.1%~0.5%	3695	0.9946	0.9291	0.9833	0.9091	0.9430
	0.5%~1%	5229	0.9927	0.8797	0.9739	0.8919	0.9288
	1%~10%	49481	0.9879	0.9373	0.9772	0.9446	0.9603
	10%~20%	22056	0.9842	0.9642	0.9823	0.9751	0.9787
	20%~30%	17336	0.9849	0.9679	0.9847	0.9828	0.9837
	30%~40%	15352	0.9844	0.9664	0.9847	0.9839	0.9843
	40%~50%	13355	0.9844	0.9643	0.9846	0.9843	0.9845
	Overall	126504	0.9865	0.9671	0.9842	0.9768	0.9804

Models' performance on 1KGP/EAS cohort

Table S34. STICI's performance on the East Asian cohort of 1KGP dataset

	MAF	#SNPs	Accuracy	r^2	Precision	Recall	F_1
5% Missing	**0.1%~0.5%**	**3486**	0.9792	0.8365	0.7288	0.6255	0.6617
	**0.5%~1%**	**4236**	0.9796	0.8892	0.7952	0.7184	0.7478
	**1%~10%**	**39158**	0.9643	0.9074	0.8577	0.8438	0.8504
	**10%~20%**	**19749**	0.9353	0.9024	0.8961	0.8884	0.8922
	**20%~30%**	**14357**	0.9195	0.8870	0.9036	0.9015	0.9025
	**30%~40%**	**13453**	0.9083	0.8674	0.9043	0.9020	0.9029
	**40%~50%**	**12864**	0.9059	0.8557	0.9053	0.9051	0.9050
	**Overall**	**107303**	0.9400	0.9074	0.9002	0.8939	0.8970
15% Missing	**MAF**	**#SNPs**	**Accuracy**	**r^2**	**Precision**	**Recall**	**F_1**
	**0.1%~0.5%**	**3486**	0.9796	0.8430	0.7387	0.6381	0.6731
	**0.5%~1%**	**4236**	0.9792	0.8826	0.7950	0.7217	0.7502
	**1%~10%**	**39158**	0.9621	0.8953	0.8582	0.8369	0.8468
	**10%~20%**	**19749**	0.9353	0.9009	0.8985	0.8904	0.8944
	**20%~30%**	**14357**	0.9212	0.8854	0.9071	0.9046	0.9058
	**30%~40%**	**13453**	0.9118	0.8675	0.9084	0.9064	0.9073
	**40%~50%**	**12864**	0.9103	0.8575	0.9098	0.9096	0.9095
	**Overall**	**107303**	0.9404	0.9046	0.9038	0.8965	0.9001
25% Missing	**MAF**	**#SNPs**	**Accuracy**	**r^2**	**Precision**	**Recall**	**F_1**
	**0.1%~0.5%**	**3486**	0.9797	0.8411	0.7540	0.6274	0.6673
	**0.5%~1%**	**4236**	0.9772	0.8592	0.7959	0.6813	0.7216
	**1%~10%**	**39158**	0.9596	0.8810	0.8619	0.8233	0.8410
	**10%~20%**	**19749**	0.9347	0.8970	0.9012	0.8912	0.8961
	**20%~30%**	**14357**	0.9231	0.8833	0.9107	0.9079	0.9093
	**30%~40%**	**13453**	0.9159	0.8683	0.9130	0.9112	0.9120
	**40%~50%**	**12864**	0.9158	0.8617	0.9153	0.9152	0.9151
	**Overall**	**107303**	0.9407	0.9010	0.9082	0.8981	0.9031
50% Missing	**MAF**	**#SNPs**	**Accuracy**	**r^2**	**Precision**	**Recall**	**F_1**
	**0.1%~0.5%**	**3486**	0.9820	0.8628	0.8077	0.6412	0.6901
	**0.5%~1%**	**4236**	0.9783	0.8626	0.8301	0.6756	0.7265
	**1%~10%**	**39158**	0.9572	0.8654	0.8786	0.8022	0.8354
	**10%~20%**	**19749**	0.9297	0.8827	0.9037	0.8838	0.8933
	**20%~30%**	**14357**	0.9206	0.8718	0.9108	0.9065	0.9086
	**30%~40%**	**13453**	0.9174	0.8631	0.9149	0.9143	0.9146
	**40%~50%**	**12864**	0.9195	0.8622	0.9192	0.9191	0.9191
	**Overall**	**107303**	0.9394	0.8930	0.9129	0.8969	0.9046

Table S35. GenoBERT's performance on the East Asian cohort of 1KGP dataset

	MAF	#SNPs	Accuracy	r^2	Precision	Recall	F_1
5% Missing	0.1%~0.5%	3486	0.9997	0.9970	0.9970	0.9970	0.9970
	0.5%~1%	4236	0.9998	0.9976	0.9974	0.9986	0.9980
	1%~10%	39158	0.9991	0.9961	0.9969	0.9970	0.9970
	10%~20%	19749	0.9976	0.9954	0.9975	0.9961	0.9968
	20%~30%	14357	0.9978	0.9953	0.9978	0.9974	0.9976
	30%~40%	13453	0.9975	0.9946	0.9975	0.9974	0.9974
	40%~50%	12864	0.9978	0.9947	0.9978	0.9978	0.9978
	Overall	107303	0.9983	0.9962	0.9979	0.9973	0.9976
15% Missing	MAF	#SNPs	Accuracy	r^2	Precision	Recall	F_1
	0.1%~0.5%	3486	0.9996	0.9958	0.9968	0.9947	0.9958
	0.5%~1%	4236	0.9993	0.9941	0.9968	0.9914	0.9941
	1%~10%	39158	0.9963	0.9856	0.9941	0.9835	0.9887
	10%~20%	19749	0.9950	0.9898	0.9949	0.9921	0.9935
	20%~30%	14357	0.9950	0.9898	0.9951	0.9943	0.9947
	30%~40%	13453	0.9951	0.9890	0.9952	0.9950	0.9951
	40%~50%	12864	0.9953	0.9889	0.9953	0.9953	0.9953
	Overall	107303	0.9958	0.9907	0.9954	0.9932	0.9943
25% Missing	MAF	#SNPs	Accuracy	r^2	Precision	Recall	F_1
	0.1%~0.5%	3486	0.9965	0.9669	0.9953	0.9271	0.9586
	0.5%~1%	4236	0.9943	0.9556	0.9934	0.9090	0.9471
	1%~10%	39158	0.9922	0.9708	0.9900	0.9632	0.9762
	10%~20%	19749	0.9917	0.9832	0.9911	0.9870	0.9891
	20%~30%	14357	0.9921	0.9841	0.9919	0.9911	0.9915
	30%~40%	13453	0.9921	0.9831	0.9922	0.9920	0.9921
	40%~50%	12864	0.9922	0.9823	0.9923	0.9922	0.9922
	Overall	107303	0.9923	0.9833	0.9919	0.9872	0.9895
50% Missing	MAF	#SNPs	Accuracy	r^2	Precision	Recall	F_1
	0.1%~0.5%	3486	0.9956	0.9561	0.9877	0.9137	0.9475
	0.5%~1%	4236	0.9917	0.9350	0.9836	0.8734	0.9212
	1%~10%	39158	0.9849	0.9436	0.9785	0.9301	0.9529
	10%~20%	19749	0.9811	0.9617	0.9798	0.9705	0.9750
	20%~30%	14357	0.9817	0.9629	0.9815	0.9793	0.9804
	30%~40%	13453	0.9820	0.9616	0.9821	0.9817	0.9819
	40%~50%	12864	0.9820	0.9596	0.9820	0.9820	0.9820
	Overall	107303	0.9837	0.9645	0.9821	0.9732	0.9776

Datasets specifications

Table S36. provides the summary and specifications of the LOS dataset.

Table S36. Specification for the LOS dataset

	Overall	Female	Male	p-value	African American	Caucasian	p-value
**#Samples (%)**	7675	3611(47.05)	4062 (52.93)		3103 (40.43)	4405 (57.39)	
**Age, yr (mean (SD))**	43.75 (14.40)	44.97 (15.54)	42.66 (13.23)	<.001	44.41 (13.14)	43.45 (15.19)	<.001
**Weight, kg (mean (SD))**	79.37 (19.46)	74.67 (20.26)	83.54 (17.72)	<.001	83.82 (20.48)	76.67 (18.19)	<.001

Table S37. provides the summary and specifications for both datasets after preprocessing steps:

Table S37. Specification of cohort composition after preprocessing for the LOS and 1KGP datasets.

	#Samples								
**Training**	6001	3522	2479	2035	417	410	404	393	411
**Validation**	749	440	309	250	51	50	50	49	50
**Test**	754	442	312	263	54	53	52	50	54
**#SNPs**	257352	188399	307599	241113	120577	199604	173237	126504	107303
**Population**	**ALL**	**CA**	**AA**	**ALL**	**EUR**	**AFR**	**AMR**	**SAS**	**EAS**
**Dataset**		**LOS**				**1KGP**			

Shown are the numbers of samples in the training, validation, and test splits, along with the number of retained SNPs, stratified by population group.
